# Single Cu Atoms Anchored Energetic COFs as Combustion Catalytic Promoters toward Rapid and Concentrated Thermal Decomposition of Ammonium Perchlorate

**DOI:** 10.1002/advs.202501761

**Published:** 2025-05-14

**Authors:** Meng Zhang, Shan Wang, Xu‐Yang Wang, Zhi‐Peng Wu, Bai‐Suo Ding, Li Yang, Wen‐Chao Tong, Qinglang Ma, Qian‐You Wang

**Affiliations:** ^1^ Frontiers Science Center for High Energy Material Advanced Research Institute of Multidisciplinary Science Beijing Institute of Technology Beijing 100081 China; ^2^ Science and Technology on Applied Physical Chemistry Laboratory Shaanxi Applied Physics‐Chemistry Research Institute Xi'an Shaanxi 710061 China; ^3^ School of Chemistry and Chemical Engineering Beijing Institute of Technology Beijing 100081 China; ^4^ Key Laboratory of Explosion Science and Technology of China Beijing Institute of Technology Beijing 100081 China; ^5^ Chongqing Advanced Materials Institute Chongqing 408107 China; ^6^ Zhengzhou Academy of Intelligent Technology Beijing Institute of Technology Zhengzhou 450000 China

**Keywords:** ammonium perchlorate, combustion catalytic promoters, covalent organic frameworks, single Cu atoms

## Abstract

Achieving a synergistic, rapid, and concentrated energy release process of ammonium perchlorate (AP) is of vital significance for boosting the thrust of composite solid propellants. However, conventional catalytic promoters often exhibit suboptimal catalytic kinetics due to inefficient utilization of active sites. Herein, atomically dispersed Cu‐coordinated covalent organic frameworks (COFs)‐based catalytic promoters are reported, decorating with energetic anion groups. The highly accessible single Cu sites confined in the COFs significantly contribute to the raid and concentrated energy release of AP, yielding a sharply narrowed decomposition peak at 341.3 °C with a peak width of only 9 °C. This performance is markedly superior to the dispersed exothermic process of raw AP (410.3 °C with a peak width of 30 °C). Additionally, the energetic COF promoter considerably increased the output energy via collaboratively promoting the chemical energy release of AP and its own decomposition. Correlating in situ spectroscopies and theoretical calculations reveals that the incorporated anionic groups effectively modulate the local electronic structure of the Cu sites. This alteration promotes the key step of cleavage of Cl─O bonds in AP intermediates to produce reactive oxygen species and also boosts the oxidation of NH_3_ to high‐valence nitrogen oxides, thereby accelerating the combustion reaction kinetic.

## Graphical abstract

Introducing energetic Cu‐based metal salts into the covalent organic frameworks (COFs) structure, Cu is in single‐atom form anchored to the framework, with energetic anions confined in the COFs structure. The synergistic interaction between single Cu atoms and the anions accelerates the reaction kinetics of ammonium perchlorate (AP) decomposition, enabling a fast and concentrated energy release.

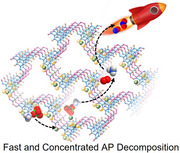

## 1 Introduction

As a key propulsion energy source of motors, solid propellant is critical to the missile defense and space exploration systems.^[^ ^1^, ^2^, ^3^ ^]^ Ammonium perchlorate (AP), the most widely used oxidant in composite solid propellants, accounts for ≈ 70% of the solid mass and directly determines the overall combustion performance of solid rocket engines.^[^ ^4^, ^5^, ^6^, ^7^, ^8^ ^]^ However, achieving fast and complete energy release of AP is challenging because the thermal decomposition process of AP occurs at relatively high degradation temperature, as well as involves several stages. Although various metal catalytic promoters, such as metal nanoparticles, transition metal oxides, organometallic coordination complexes, and carbon materials, have been developed to advance the chemical propulsion of AP,^[^ ^9^, ^10^, ^11^ ^]^ they still show unsatisfying combustion performance due to the low atomic utilization of active sites. For example, recent studies show that metal catalytic promoters could reduce the degradation temperature of AP.^[^ ^12^, ^13^, ^14^, ^15^, ^16^ ^]^ Unfortunately, most of them usually showed a broad decomposition peak, implying the relatively sluggish AP combustion kinetic and diffused heat release process, which is unfavorable for realizing controllable and reliable thrust of composite solid propellants.^[^ ^17^, ^18^ ^]^ Moreover, the presence of energy‐free metal catalytic promoters inevitably compromised the total released energy of solid propellants.^[^ ^19^, ^20^, ^21^ ^]^ Thus, the development of advanced AP decomposition promoters with high atomic utilization efficiency and energetic characteristics is desirable to achieve the goal of controllable and enhanced energy release processes in solid propellants.^[^ ^22^, ^23^ ^]^

Covalent organic frameworks (COFs) are polymeric crystalline materials synthesized through the reticulation of organic building units connected by covalent linkage.^[^ ^24^, ^25^, ^26^ ^]^ COFs possess chemical diversity, tunable open channels, and high reactant accessibility, making them a subject of considerable research interest.^[^ ^27^, ^28^, ^29^, ^30^ ^]^ Immobilizing single metal sites into COFs with adjustable network structures has been demonstrated to be a versatile method to reach innovative catalysts, which could exhibit the utmost atom utilization efficiency.^[^ ^31^, ^32^ ^]^ Moreover, the highly ordered structure of porous COFs facilitates efficient electron and mass transport, thereby accelerating energy release rates.^[^ ^33^, ^34^, ^35^ ^]^ In addition, the rational design of the energetic ligands as building units in COF‐based catalytic promoters can boost the energy level of the system.

In this work, we report the design and synthesis of novel single Cu atoms anchored COFs‐based catalytic promoters. The readily synthesized COFs (Tp‐CBH‐Cu‐ClO_4_ and Tp‐CBH‐Cu‐N(NO_2_)_2_) simultaneously contain single Cu atoms with Cu‐N/O atomic coordination and energetic anion groups in the pores. When evaluating their catalytic ability on the thermal degradation of AP, we found that Tp‐CBH‐Cu‐ClO_4_ and Tp‐CBH‐Cu‐N(NO_2_)_2_ effectively promote the thermal decomposition of AP, achieving a single‐step decomposition of AP at a low temperature of 351.3 and 341.3 °C, respectively, compared to the multi‐step degradation of pure AP with high decomposition temperature of 410.3 °C. Impressively, the exothermic peaks are exceptionally narrow, demonstrating the desirable rapid and concentrated decomposition process of AP, and position Tp‐CBH‐Cu‐N(NO_2_)_2_ as one of the fastest Cu‐based catalytic promoters. In addition, the energetic COF promoter is endowed with considerable energy release via collaboratively promoting the chemical energy release of AP and its own decomposition. In situ thermogravimetric‐fourier transform infrared‐gas chromatography mass spectrometry (TG‐FTIR‐GC/MS) analysis, combined with density functional theory (DFT) calculations, demonstrates that the single Cu atoms confined in the structure of COF as the active sites and the anionic groups incorporated in the framework effectively tailor the AP decomposition efficiency. Consequently, the unique structure promotes the critical stage of HClO_4_ dissociation to produce reactive oxygen species and facilitates the oxidation of NH_3_ to high‐valence nitrogen oxides, ultimately showing great advances in fast and concentrated thermal decomposition of AP. These findings provide valuable atomic‐scale insights into the ingenious design of advanced catalytic promoters for solid propellants.

## 2 Results and Discussion

**Figure 1:**
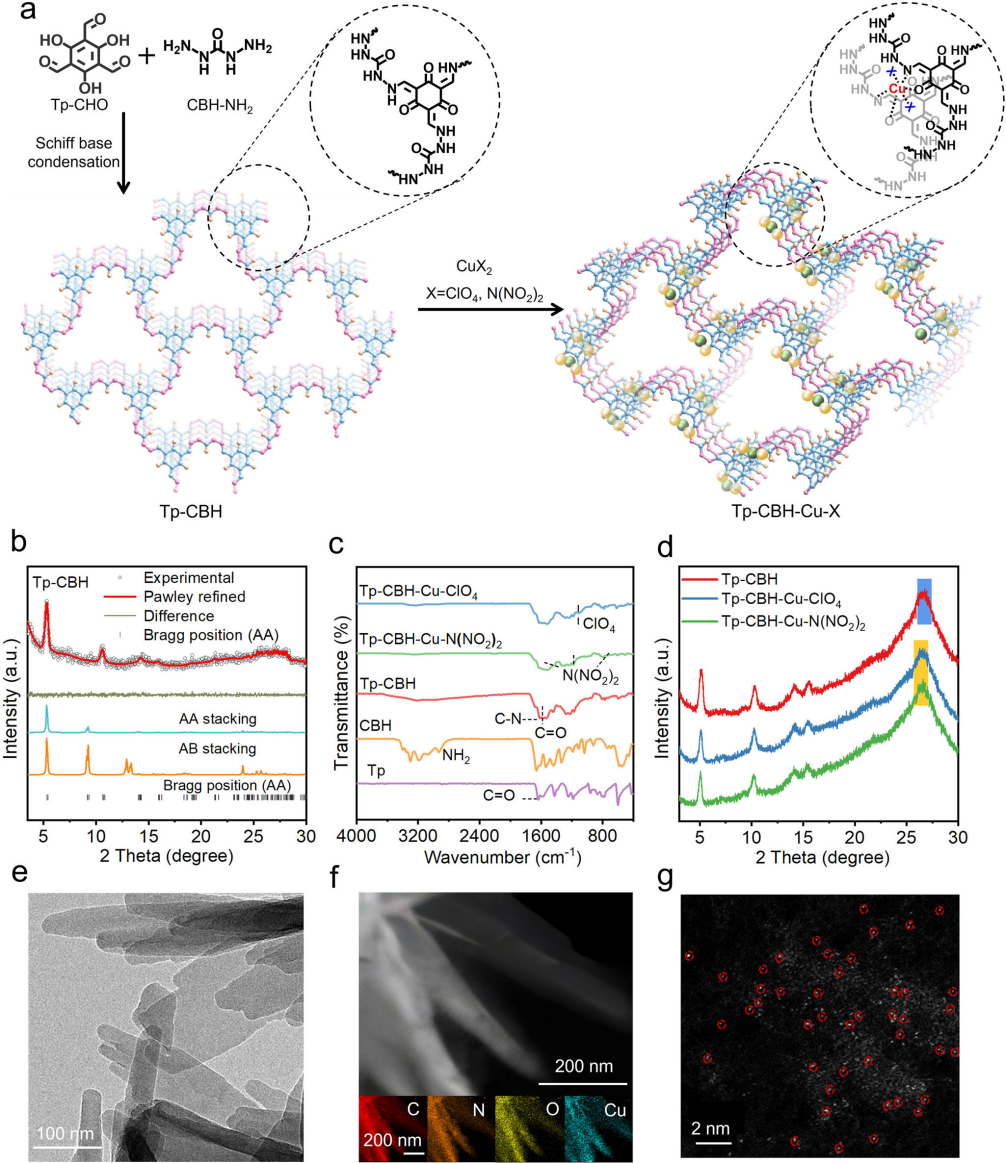
a) Schematic of the synthesis and structure of Tp‐CBH, Tp‐CBH‐Cu‐ClO_4,_ and Tp‐CBH‐Cu‐N(NO_2_)_2_. b) Experimental, pawley refined, and simulated PXRD patterns (AA stacking and AB stacking mode) of Tp‐CBH. c) FT‐IR spectra of Tp, CBH, Tp‐CBH, Tp‐CBH‐Cu‐ClO_4,_ and Tp‐CBH‐Cu‐N(NO_2_)_2_. d) PXRD patterns of Tp‐CBH, Tp‐CBH‐Cu‐ClO_4,_ and Tp‐CBH‐Cu‐N(NO_2_)_2_. e) TEM image, f) Element mapping, and g) Atomic‐resolution HAADF‐STEM image of Tp‐CBH‐Cu‐N(NO_2_)_2_.

A crystalline COF precursor, Tp‐CBH, was first synthesized by condensing triformyl phloroglucinol (Tp‐CHO) and carbohydrazide (CBH‐NH_2_), (**Figure** **1**a). Powder X‐ray diffraction (PXRD) measurement was carried out to confirm the crystallinity of Tp‐CBH. Pawley refinement was conducted with the experimental PXRD pattern of Tp‐CBH in the space group of *P‐6m* to yield unit cell parameters of *a*  = 19.043 Å,  *b*  =  3.556  Å, *c*  =  19.018  Å, *α*  =  *γ*  =  90°, and  *β* =  120° with profile residual factor *R*_p_  =  2.89% and weighted profile residual factor *R*_wp_  =  3.83%. The diffraction peaks at 5.34°, 10.64°, 14.38°, and 26.62° were assigned to the (100), (010), (200), and (001) lattice planes, respectively (Figure 1b). The experimental PXRD pattern of Tp‐CBH matched well with the simulated structure, where Tp‐CBH crystallized in an eclipsed AA stacking arrangement.

The chemical structures of the Tp‐CBH COF were then confirmed by Fourier‐transform infrared (FT‐IR) spectroscopy and ^13^C solid‐state nuclear magnetic resonance (^13^C *ss*NMR) spectroscopy. In the FT‐IR spectrum of Tp‐CBH, the typical signals for the amine group of CBH‐NH_2_ ( 2925–3403 cm^−1^) and the aldehyde group of Tp‐CHO (1646 cm^−1^) disappeared, while the new characteristic stretching peaks of the C═O (1589 cm^−1^) and C─N (1610 cm^−1^) groups were observed, indicating the successful condensation between the amino and aldehyde groups to form a β‐ketoenamine structure (Figure 1c). In the solid‐state ^13^C *ss*NMR spectra, Tp‐CBH showed a distinct signal near 192 ppm, which corresponds to the C═O carbon atoms. Furthermore, two peaks at ≈ 142 and 99 ppm are linked to the C═C carbons, providing further evidence for the formation of β‐ketoenamine linked COF (Figure S2, Supporting Information). The N_2_ sorption isotherm of Tp‐CBH was measured at 77 K (Figure S3, Supporting Information), giving a Brunauer‐Emmett‐Teller (BET) surface area of 179 m^2^ g^−1^, with an average pore size of 1.5 nm as estimated by the nonlocal density functional theory (NLDFT) model (Figure S4, Supporting Information).

Subsequent treatment of Tp‐CBH with Cu(ClO_4_)_2_ or CuN[(NO_2_)]_2_ in ethanol solution produced Tp‐CBH‐Cu‐ClO_4_ and Tp‐CBH‐Cu‐N(NO_2_)_2_, respectively. After implanting Cu atoms, the FT‐IR spectra of Tp‐CBH‐Cu‐ClO_4_ and Tp‐CBH‐Cu‐N(NO_2_)_2_ preserve the characteristic functional groups of Tp‐CBH, revealing that the frameworks were well maintained. Meanwhile, the stretching band of ClO_4_^−^ functional groups at 1114 cm^−1^ and the stretching vibrations of ‐NO₂ moieties from the N(NO_2_)_2_^−^ anions at 1326, 1009, and 756 cm^−1^ were found in Tp‐CBH‐Cu‐ClO_4_ and Tp‐CBH‐Cu‐N(NO_2_)_2_, respectively (Figure 1c). For the Tp‐CBH‐Cu‐ClO_4_ and Tp‐CBH‐Cu‐N(NO_2_)_2_, the PXRD patterns of resultant samples are similar to those of Tp‐CBH, indicating that the crystalline structure was not destroyed after the coordination of Cu ions. The peak of (001) plane at 26.62° shifted towards lower angles, suggesting that the copper ions were most probably positioned between the adjacent layers, resulting in enhanced interlayer spacing (Figure 1d).^[^ ^36^, ^37^ ^]^ The refined structural models further validated the expanded interlayer spacing induced by copper ion incorporation (Figure S5). Besides, no diffraction peak of metal copper displayed in the PXRD pattern of Tp‐CBH‐Cu‐ClO_4_ and Tp‐CBH‐Cu‐N(NO_2_)_2_, indicating that Cu ions existed in the coordinated structure within the COFs. N_2_ sorption isotherm tests revealed that after post‐modification with Cu(ClO_4_)_2_ or Cu[N(NO_2_)_2_], the BET of Tp‐CBH‐Cu‐ClO_4_ and Tp‐CBH‐Cu‐N(NO_2_)_2_ decreased to 67 and 58 m^2^ g^−1^, respectively, and exhibited non‐porous characteristics, which might be attributed to the copper salts blocking the pore structure of Tp‐CBH. These results indicated the successful incorporation of the copper salt within the Tp‐CBH framework.

The morphologies of Tp‐CBH, Tp‐CBH‐Cu‐ClO_4,_ and Tp‐CBH‐Cu‐N(NO_2_)_2_ were visualized by electron microscopy. Scanning electron microscopy (SEM) and transmission electron microscopy (TEM) images of all materials revealed rod‐shaped structures without clear formation of Cu nanoparticles (Figure 1e; Figures S6–S10, Supporting Information). Energy dispersive spectroscopy (EDS) measurements showed that C, N, O, and Cu elements were evenly dispersed over the Tp‐CBH‐Cu‐ClO_4_ and Tp‐CBH‐Cu‐N(NO_2_)_2_ (Figure 1f; Figures S6–S10, Supporting Information). Furthermore, the aberration‐corrected high‐angle annular dark‐field scanning transmission electron microscopy (AC‐HAADF‐STEM) images clearly showed abundant bright spots, which were assigned to Cu atoms, confirming that Cu element presented atomically dispersion on the COF substrate (Figure 1g; Figure S11, Supporting Information).

**Figure 2:**
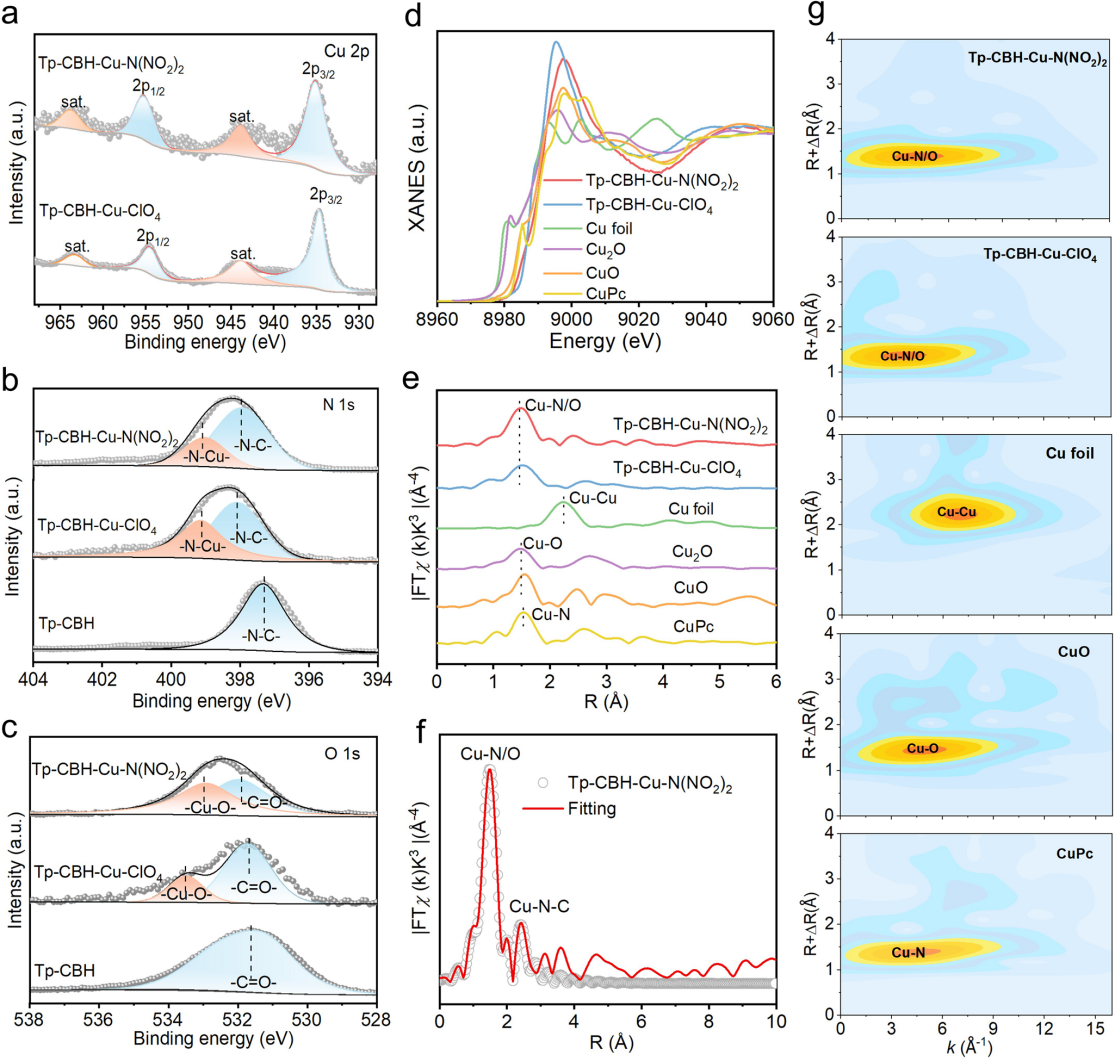
XPS spectra of a) Cu 2p in Tp‐CBH‐Cu‐ClO_4_ and Tp‐CBH‐Cu‐N(NO_2_)_2,_ b) N 1s and c) O 1s in Tp‐CBH, Tp‐CBH‐Cu‐ClO_4_ and Tp‐CBH‐Cu‐N(NO_2_)_2_. d) XANES and e) EXAFS spectra of Tp‐CBH‐Cu‐N(NO_2_)_2_, Tp‐CBH‐Cu‐ClO_4_, and the reference samples. f) Fitting curves for Tp‐CBH‐Cu‐N(NO_2_)_2_ in the R‐space. g) WT‐EXAFS contour plots of Tp‐CBH‐Cu‐N(NO_2_)_2_, Tp‐CBH‐Cu‐ClO_4_, Cu foil, CuO, and CuPc.

To investigate the chemical state of Cu atoms in Tp‐CBH‐Cu‐ClO_4_ and Tp‐CBH‐Cu‐N(NO_2_)_2_, X‐ray photoelectron spectroscopy (XPS) was employed. Apparently, in the XPS survey spectra, characteristic peaks of Cu appeared in Tp‐CBH‐Cu‐ClO_4_ and Tp‐CBH‐Cu‐N(NO_2_)_2_, which were absent in Tp‐CBH (Figure S12, Supporting Information). The high‐resolution XPS spectra of Cu 2p for Tp‐CBH‐Cu‐ClO_4_ and Tp‐CBH‐Cu‐N(NO_2_)_2_ showed peaks at 954.75 and 934.85 eV, which can be assigned to Cu 2p_1/2_ and Cu 2p_3/2_, respectively, indicating that Cu species have an oxidation state of ≈+2 (**Figure** **2**a). In the high‐resolution N 1s spectra, a new peak at 399.4 eV appeared after the introduction of Cu species, corresponding to Cu‐N bonding (Figure 2b). A positive upshift of N‐C binding energy occurs in Tp‐CBH‐Cu‐ClO_4_ and Tp‐CBH‐Cu‐N(NO_2_)_2_ when compared with that of Tp‐CBH, suggesting stronger electronic interactions between N and Cu atoms. Similarly, in the O 1s spectra, the C═O binding energy in Tp‐CBH‐Cu‐ClO_4_ and Tp‐CBH‐Cu‐N(NO_2_)_2_ shifts toward higher binding energies than that of Tp‐CBH, accompanied by new peaks of Cu‐O bonding at 533.7 and 533.2 eV for Tp‐CBH‐Cu‐ClO_4_ and Tp‐CBH‐Cu‐N(NO_2_)_2_, respectively (Figure 2c). These results indicate that Cu^2+^ is firmly anchored to the framework of Tp‐CBH through coordination with nitrogen and oxygen atoms.

Synchrotron‐based X‐ray absorption spectroscopy (XAS) experiments were carried out to further probe the atomic structures in terms of valance states and coordination environment of Cu species in Tp‐CBH‐Cu‐ClO_4_ and Tp‐CBH‐Cu‐N(NO_2_)_2_. Figure 2d shows the X‐ray absorption near‐edge structure (XANES) curves of the Tp‐CBH‐Cu‐ClO_4_, Tp‐CBH‐Cu‐N(NO_2_)_2_, and reference samples at the Cu K‐edge. The white‐line peak for Tp‐CBH‐Cu‐ClO_4_ and Tp‐CBH‐Cu‐N(NO_2_)_2_ is close to that of CuO, suggesting that the oxidation state of the Cu species is ≈ +2. This result is also consistent with the XPS analysis. The fourier transform of the extended X‐ray absorption fine structure (EXAFS) spectra of Tp‐CBH‐Cu‐ClO_4_ and Tp‐CBH‐Cu‐N(NO_2_)_2_ display a major peak at ≈ 1.47 Å (Figure 2e), which is close to peaks of Cu‐O at 1.49 Å for CuO and Cu‐N at 1.51 Å for CuPc, suggesting that the Cu atom is coordinated with N/O atoms.^[^ ^38^ ^]^ Quantitative structural parameters for Cu in Tp‐CBH‐Cu‐ClO_4_ and Tp‐CBH‐Cu‐N(NO_2_)_2_ were determined by using FT‐EXAFS fitting spectrum (Table S3, Supporting Information). For Tp‐CBH‐Cu‐ClO_4_, the fitting results showed the coordination numbers of Cu consisting of 2.1 N atoms at a bond length of 1.90 Å and 3.9 O atoms at a bond length of 1.97 Å (Figure S13, Supporting Information). Similarly, the Cu species in Tp‐CBH‐Cu‐N(NO_2_)_2_ coordinated with 2.1 N atoms at a bond length of 1.99 Å and 3.5 O atoms at a bond length of 1.90 Å (Figure 2f). To reveal more atomic structure information of Tp‐CBH‐Cu‐ClO_4_ and Tp‐CBH‐Cu‐N(NO_2_)_2_, wavelet‐transform (WT) analysis was carried out to provide powerful resolution in both k and R spaces (Figure 2g). Taking Tp‐CBH‐Cu‐N(NO_2_)_2_ as an example, the maximum intensity of WT at 4.3 Å^−1^ (k space) is adequately resolved at 1.4 Å (R space), which is ascribed to the Cu‐N/O coordination structure. The absence of Cu‐Cu maximum intensity at 6.9 Å^−1^ (k space) and 2.2 Å^−1^ (R space) corresponds to the reference samples of Cu‐foil further confirms the incorporation of Cu single atoms into Tp‐CBH network. The inductively coupled plasma mass spectrometry (ICP‐MS) shows that the Cu content in Tp‐CBH‐Cu‐ClO_4_ and Tp‐CBH‐Cu‐N(NO_2_)_2_ was ≈ 6.75 and 5.2 wt.%, respectively (Table S2, Supporting Information).

The thermal decomposition behavior of the Tp‐CBH‐Cu‐ClO_4_ and Tp‐CBH‐Cu‐N(NO_2_)_2_ was evaluated by thermogravimetric analysis (TGA) in an inert N_2_ atmosphere (Figure S14a, Supporting Information). A major weight loss is started in the range of 190–200 °C, corresponding to the collapse of the COF skeleton. Upon addition of 10 wt.% Tp‐CBH, Tp‐CBH‐Cu‐ClO_4_, and Tp‐CBH‐Cu‐N(NO_2_)_2_ as AP decomposition promoters, the drastic weight loss of mixtures initiated at ≈ 260 °C (Figure S14b, Supporting Information). Compared with pure COF, the decomposition phenomenon of mixtures shifted to higher temperature. This result indicated that the presence of AP could stabilize the COF materials, preventing COF decomposition. Meanwhile, the decomposition temperature of these mixtures are lower than that of raw AP (279.4 °C), showing a crucial role of COF promoters in the catalysis of AP. The COF/AP mixtures exhibited a nearly 100% weight loss similar to pure AP, indicating that the thermal degradation of COF and AP is integrated. Additionally, we tested the PXRD of pristine Tp‐CBH‐Cu‐N(NO_2_)_2_ and Tp‐CBH‐Cu‐N(NO_2_)_2_ with a mixing mass of 50% AP before and after heating at 350 °C for 5 min. The results showed that the characteristic diffraction peaks of Tp‐CBH‐Cu‐N(NO_2_)_2_ with a mixing mass of 50% AP following thermal exposure remained intact, while those of pure Tp‐CBH‐Cu‐N(NO_2_)_2_ had disappeared (Figure S15, Supporting Information). This further proves that the presence of AP stabilizes Tp‐CBH‐Cu‐N(NO_2_)_2_, enabling it to sustain catalytic activity throughout AP decomposition. Based on the above thermal analysis, Tp‐CBH, Tp‐CBH‐Cu‐ClO_4_, and Tp‐CBH‐Cu‐N(NO_2_)_2_ could serve as effective catalytic promoters for the thermal degradation of AP.

**Figure 3:**
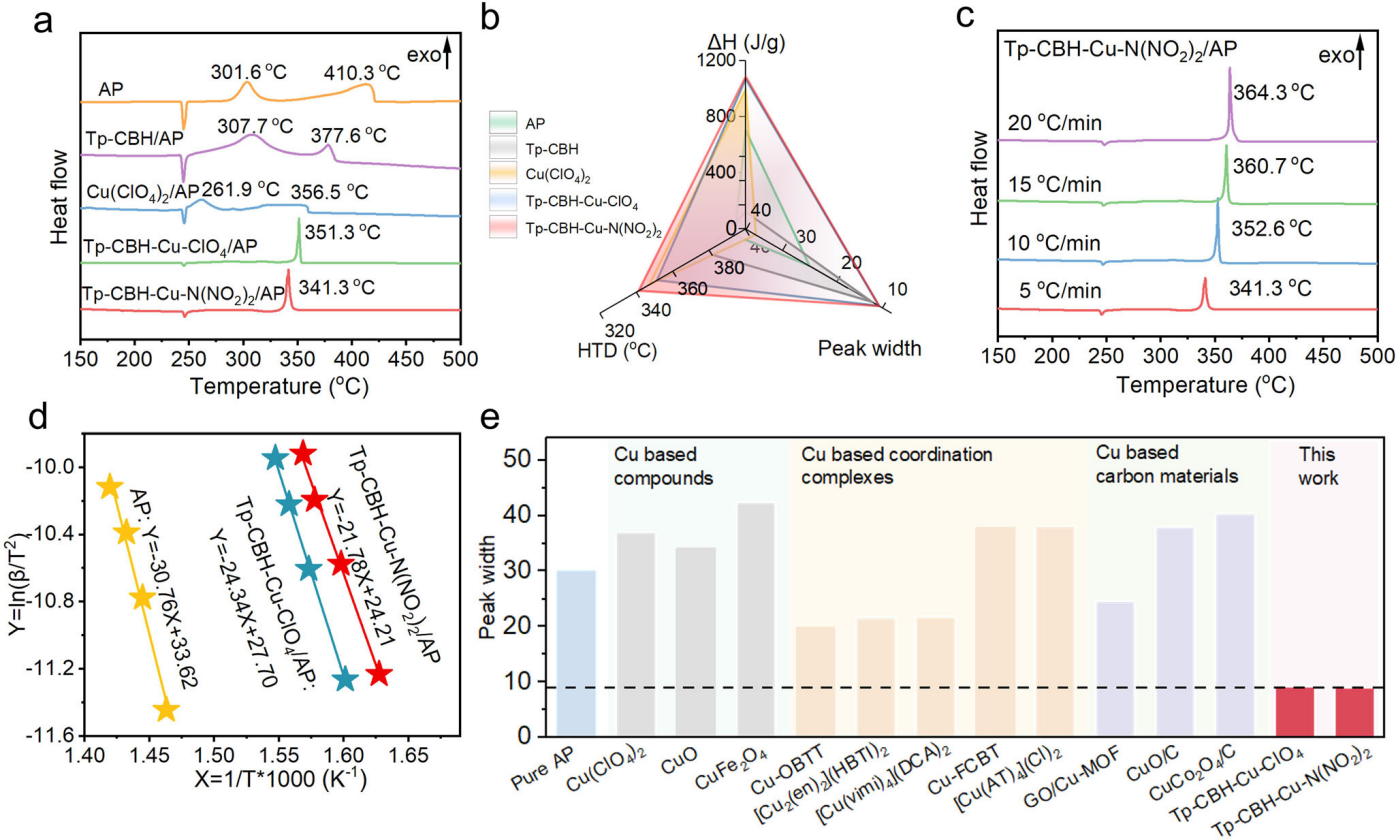
a) DSC curves for AP and various catalysts/AP mixtures at 5 °C min^−1^. b) Comparison of the HTD, peak width and ΔH for Tp‐CBH, Tp‐CBH‐Cu‐ClO_4_, and Tp‐CBH‐Cu‐N(NO_2_)_2_. c) DSC curves of Tp‐CBH‐Cu‐N(NO_2_)_2_/AP mixtures at different heating rates. d) Fitted Kissinger activation energies of AP and corresponding mixtures. e) Comparison of the peak width of Tp‐CBH, Tp‐CBH‐Cu‐ClO_4_, and Tp‐CBH‐Cu‐N(NO_2_)_2_ with other reported Cu‐based catalyst.

The catalytic effects of Tp‐CBH‐Cu‐ClO_4_ and Tp‐CBH‐Cu‐N(NO_2_)_2_ on AP decomposition were investigated by differential scanning calorimetry (DSC) in a N_2_ atmosphere in the range of 150–500 °C, together with the precursors Tp‐CBH and Cu(ClO_4_)_2_ as reference materials. We first screened catalyst loadings of 5%, 10%, and 15% (Figure S16, Supporting Information). Based on optimization, a 10% catalyst loading was determined to be the optimal amount. As shown in **Figure** **3**a for the DSC curves, we observed a prominent endothermic peak of pure AP at ≈ 243.6 °C, indicating the crystalline phase transformation from orthorhombic to cubic structure. This endothermic phase transition peak had almost no shift and exhibited a similar profile with Tp‐CBH‐Cu‐ClO_4_ and Tp‐CBH‐Cu‐N(NO_2_)_2_. As the temperature increased, two exothermic peaks of AP were observed, corresponding to the low‐temperature decomposition (LTD) at 301.6 °C and high‐temperature decomposition (HTD) at 410.3 °C. Notably, upon the addition of Tp‐CBH‐Cu‐ClO_4_ and Tp‐CBH‐Cu‐N(NO_2_)_2_, only one exothermic peak was observed during the whole AP decomposition, indicating that the LTD and HTD peaks of AP merge into one exothermic peak. This result suggested that the introduction of Cu sites within the COF catalysts suppressed the LTD stage of AP and advanced the HTD exothermic peak, changing the thermal decomposition behavior of AP and concentrating the energy release of AP. The exothermic peaks of Tp‐CBH‐Cu‐ClO_4_ and Tp‐CBH‐Cu‐N(NO_2_)_2_ can be advanced to 351.3 and 341.3 °C, respectively, which is 59.0, and 69.0 °C lower than that of HTD stage of pure AP (410.3 °C). More importantly, the width of the exothermic peak of Tp‐CBH‐Cu‐ClO_4_ and Tp‐CBH‐Cu‐N(NO_2_)_2_ was only ≈ 9 °C, which was significantly narrower than that of ≈ 30 °C observed by the pure AP. The extremely narrow width validated that the AP decomposition reaction is a rapid and intense process. Meantime, a series of comparison experiments with different sample mass (1 and 3 mg) and atmosphere (O_2_ and N_2_) were conducted (Figure S17, Supporting Information). Referring to DSC curves, they have no impact on the narrow exothermic peak of the decomposition, highlighting that the intrinsic catalytic effects of COFs induced the rapid decomposition of AP. In addition, the influence of anions confined in framework on the thermal decomposition behavior of AP was investigated by employing Tp‐CBH‐Cu‐OAc and Tp‐CBH‐Cu‐Cl, obtained from Tp‐CBH reacted with Cu(OAc)_2_ and CuCl_2_ respectively, as references. Both showed one concentrated exothermic peak at 353.2 and 352.6 °C during AP decomposition, respectively (Figures S18–S20, Supporting Information), which further verified that the single Cu sites greatly promoted rapid and concentrated AP decomposition. Referring to DSC curves of the four single Cu sites anchored COF samples with different anions, Tp‐CBH‐Cu‐N(NO_2_)_2_ showed the lowest decomposition temperature. We deduced that ‐N(NO_2_)_2_ synergistically promoted AP decomposition.^[^ ^39^, ^40^ ^]^ A comprehensive comparison of the peak width of the exothermic peak with previously reported Cu‐based combustion catalysts, presented in Figure 3e,^[^ ^6^, ^7^, ^12^, ^13^, ^14^, ^18^, ^41^ ^]^ showcased the sharpest AP decomposition peak of single Cu atoms anchored COF catalysts, underscoring their exceptionally fast combustion rate. At the same time, the overall process is accompanied by 794, 870, 1068, and 1086 J g^−1^ heat release (ΔH) of Tp‐CBH‐Cu‐Cl, Tp‐CBH‐Cu‐OAc, Tp‐CBH‐Cu‐ClO_4_, and Tp‐CBH‐Cu‐N(NO_2_)_2_, respectively. Compared with 637.34 J g^−1^ of pure AP, the energy release level in the course of the thermal decomposition of AP by Tp‐CBH‐Cu‐Cl and Tp‐CBH‐Cu‐OAc slightly increased, while that of Tp‐CBH‐Cu‐ClO_4_ and Tp‐CBH‐Cu‐N(NO_2_)_2_ is significantly improved (Table S4), ascribing to the synergistic exothermic oxidation of the COF skeleton, energetic anionic groups, and AP. Regarding the exothermic behavior, the Tp‐CBH‐Cu‐ClO_4_ and Tp‐CBH‐Cu‐N(NO_2_)_2_ demonstrate substantial exothermic characteristics with heat release values of 323 and 391 J g^−1^, respectively (Figure S21, Supporting Information), proving significant energy output and energetic properties. Based on these data, the theoretical value of 10% Tp‐CBH‐Cu‐N(NO_2_)_2_ and 90% AP physical mixture was calculated to be 645 J g^−1^, which is significantly lower than that of experimental results 1086 J g^−1^, further confirming the effective catalytic role of COF in enhancing AP decomposition and energy release. In other words, the atomically Cu sites‐decorated COF catalyst could promote the chemical energy release of AP, but also contribute to energy release via decomposition itself.

On the contrary, the control samples of raw AP, Tp‐CBH, and Cu(ClO_4_)_2_ also exhibited two wide exothermic peaks during the thermal degradation (Figures S22–S24, Supporting Information). The HTD stage of Tp‐CBH and Cu(ClO_4_)_2_ is 26.3 and 5.2 °C higher than that of Tp‐CBH‐Cu‐ClO_4_ (Figure S25, Supporting Information), respectively, suggesting their inferior catalytic effect. Also, their broader exothermic peaks are indicative of their slower and non‐concentrated energy release process. Upon comparison of the decomposition behavior of AP with the Tp‐CBH, Cu‐ClO_4_, Tp‐CBH‐Cu‐ClO_4_, and Tp‐CBH‐Cu‐N(NO_2_)_2_ catalytic promoters, the Tp‐CBH‐Cu‐N(NO_2_)_2_ as modifier has the best catalytic effect in terms of decomposition temperature, peak width, and ΔH (Figure 3b). We concluded that the unique structural advantages endow Tp‐CBH‐Cu‐N(NO_2_)_2_ with better catalytic effect, which will be discussed later.

Furthermore, the study of the thermal decomposition kinetics of the mixtures of the COF catalysts with AP was carried out utilizing the non‐isothermal Kissinger method (**Equation (1)** and Figure 3d).^[^ ^42^, ^43^ ^]^

(1) $$\ln\left(\frac{\beta }{T_{p}^{2}}\right)=\ln\frac{AR}{E_{a}}-\frac{E_{a}}{R}\frac{1}{T_{p}}$$

where β, Tp, A, R, and *E*_a_ represent the heating rate (°C·min^−1^), the exothermic peak temperature (K), the pre‐exponential factor, the ideal gas constant (8.314 J mol^−1^·K^−1^), and the apparent activation energy (kJ·mol^−1^), respectively. The samples were heated from 150 to 500 °C at heating rates of 5, 10, 15, and 20 °C min^−1^, respectively. Based on Equation (1), the term ln (𝛽/*T*_p_^2^) varies linearly with 1/*T*_p_. E_a_ and A are obtained from the slope and the intercept, respectively, and the results are summarized in Table S5. The *E*_a_ values for Tp‐CBH‐Cu‐ClO_4_ and Tp‐CBH‐Cu‐N(NO_2_)_2_ were found to be 202.4 and 181.1 kJ mol^−1^, respectively, which are greatly lower than that of pure AP (255.8 kJ mol^−1^). The decreased E_a_ indicated that the addition of COF catalyst results in improved thermal stability and accelerated decomposition kinetics of AP.

**Figure 4:**
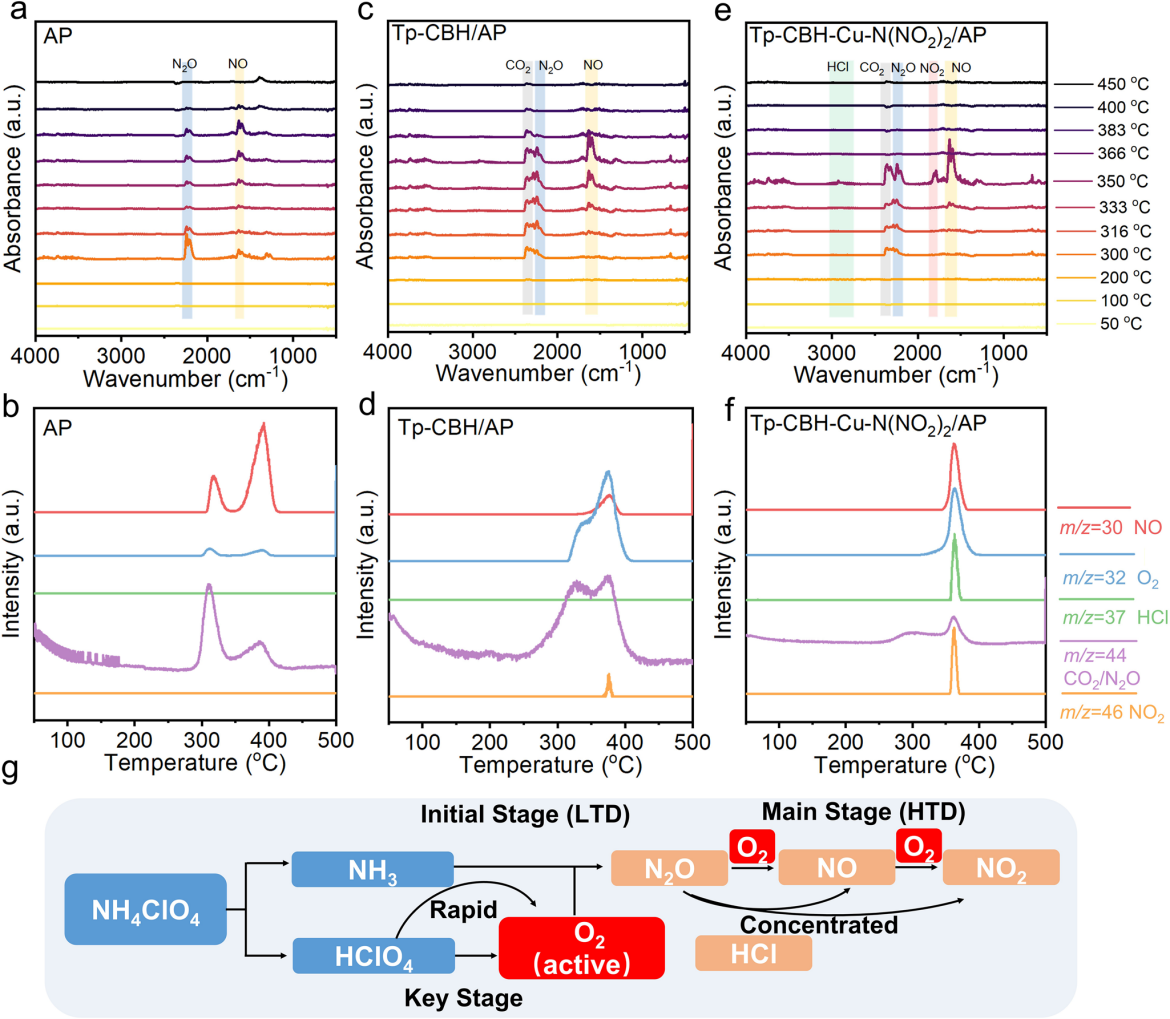
In situ TG‐FTIR‐GC/MS test results of gas production: a,b) AP; c,d) Tp‐CBH/AP; e,f) Tp‐CBH‐Cu‐N(NO_2_)_2_/AP. g) Illustration of the mechanism of the decomposition process of AP.

To gain an in‐depth understanding of the decomposition process of AP catalyzed by single Cu sites anchored COF as catalysts, a comprehensive in situ thermal analysis technology thermogravimetric‐fourier transform infrared‐gas chromatography mass spectrometry (TG‐FTIR‐GC/MS) was conducted to monitor the volatile products generated during the thermal decomposition of the AP/COF system in real‐time and study the reaction pathways. Previous studies have well established the process of AP thermal decomposition, which involves two steps: i) during the LTD stage (≈300 °C), the proton transfer from NH_4_^+^ to ClO_4_^−^, followed by the generation of intermediate products NH_3_ and HClO_4_, and the accumulation of NH_3_ on the AP surface will inhibit the continuous decomposition of AP; ii) with the increase of temperature rising to the HTD stage (≈410 °C), HClO_4_ decomposes into active oxygen species, then react with NH_3_ to form oxidized gas phase products.^[^ ^44^, ^45^ ^]^ In the gas product analysis of AP decomposition, N_2_O is typically identified as the initial oxidative decomposition product in LTD stage, while NO and NO_2_ are the main gas products in HTD satge.^[^ ^42^ ^]^ Our in situ TG‐FTIR‐GC/MS results of pure AP's degradation behavior are consistent with these reports, as it appears an N_2_O characteristic peak near 2300 cm^−1^ at ≈300 °C, then decreases with the increase of temperature, indicating that the LTD stage of AP is finished. When the temperature increases further to 350 °C, the HTD phase begins, with NO characteristic peak emerging at 1570–1680 cm^−1^, and disappearing at 400 °C, suggesting a completion of the decomposition process (**Figure** **4**a). The TG‐MS data showed similar results. During the LTD stage, the peaks of N_2_O (m/z = 44) with high intensity were extracted (Figure 4b). As the temperature rises to 350 °C, the main product had become NO. When Tp‐CBH is used as a catalyst, the TG‐FTIR showed that the gas characteristic peak of CO_2_ at 2270–2400 cm^−1^ is observed in the temperature range of 300–350 °C, which can be attributed to the decomposition of the COF structure (Figure 4c). This process is accompanied with the generation of N_2_O (2150‐2280 cm^−1^). The high‐valence NO peak (1570‐1680 cm^−1^) appeared at 333 °C, reaching a maximum value at 366 °C. TG‐MS results also showed corresponding CO_2_ (m/z = 44), O_2_ (m/z = 32), and NO (m/z = 30) signals (Figure 4d).

According to the analysis of gas products of the mixtures of Tp‐CBH‐Cu‐N(NO_2_)_2_ and AP, Cu‐anchored COF catalysts obviously changed the thermal decomposition behavior of AP. Primarily, the higher oxidation product of NO_2_ peak is observed at 1750–1860 cm^−1^. Moreover, the main decomposition process is concentrated in a narrower temperature range (300–350 °C) compared to the raw AP (300–400 °C) and Tp‐CBH (300–380 °C), proving the faster decomposition of AP (Figure 4e). Additionally, the peak intensities of nitrogen‐containing oxides (NO_x_) species are higher than the control samples. These results can be ascribed to the stronger catalytic activity of the Cu‐based COF catalysts, resulting in a higher concentration of NO_x_ compounds. Correspondingly, there is a simultaneous evolution of multiple sharp and intense gas peaks of NO (m/z = 30), O_2_ (m/z = 32), HCl (m/z = 37) and NO_2_ (m/z = 46) in the TG‐MS spectra of Tp‐CBH‐Cu‐N(NO_2_)_2_/AP mixtures, demonstrating the rapid reaction process (Figure 4f). The distinct sharp peaks of HCl and NO_2_ suggested the facilitated HClO_4_ dissociation and subsequent NH_3_ oxidation rates as catalyzed by Tp‐CBH‐Cu‐N(NO_2_)_2_. Moreover, by comparing the active O_2_ signals with raw AP, Tp‐CBH/AP, and Tp‐CBH‐Cu‐N(NO_2_)_2_/AP mixtures, we deduced that the intense O_2_ peak can oxidize NH_3_ rapidly, and eliminate the separated LTD and HTD space periods, thereby achieving a one‐step concentrated decomposition of AP instead of the multi‐step decomposition of raw AP and Tp‐CBH/AP. These results highlight that the Tp‐CBH‐Cu‐N(NO_2_)_2_ is a desirable promoter that can trigger fast decomposition of AP. Different thermal decomposition routes of AP (Figure 4g), showing that O_2_ accelerates the decomposition process of AP and leads to the occurrence of an exothermic peak.

**Figure 5:**
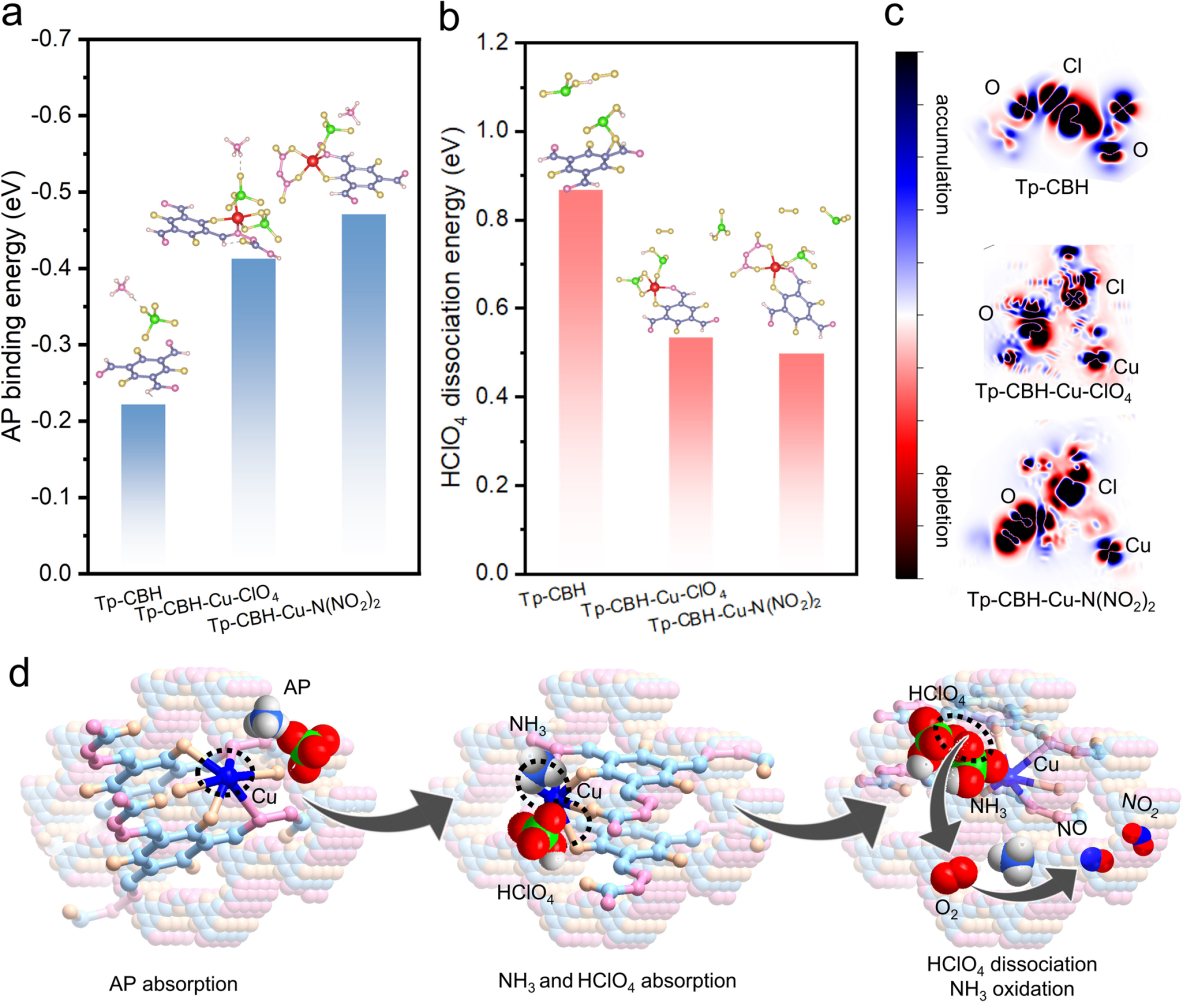
a) Comparison of AP binding energy and b) HClO_4_ dissociation energy of Tp‐CBH, Tp‐CBH‐Cu‐ClO_4_, and Tp‐CBH‐Cu‐N(NO_2_)_2_. c) Differential charge density maps during HClO_4_ adsorption in Tp‐CBH, Tp‐CBH‐Cu‐ClO_4_, and Tp‐CBH‐Cu‐N(NO_2_)_2_, the blue and red regions correspond to the charge accumulation and depletion, respectively. d) Schematic illustration of the AP thermal decomposition process.

To better understand the underlying catalytic mechanism of Cu‐anchored COF catalytic promoters on the degradation behavior of AP, we further conducted DFT calculations as implemented in the Vienna ab initio simulation package (VASP). Initially, the adsorption energies of AP on different COFs are calculated. As presented in **Figure** **5**a, the adsorption energy of AP on Tp‐CBH is only ‐0.22 eV. This value greatly enhanced after Cu anchoring into the framework. The Tp‐CBH‐Cu‐N(NO_2_)_2_ is more sensitive to the adsorption of AP via Cu‐O interaction with an adsorption energy of ‐0.47 eV than the Tp‐CBH‐Cu‐ClO4 (‐0.41 eV). The greatly increased adsorption energy indicates that AP would be more likely to adsorb and accumulate on the Cu‐decorated COF catalysts, thereby boosting the subsequent decomposition process. Considering that the adsorption of key intermediate products NH_3_ on the surface would inhibit the AP decomposition, the adsorption energy of NH_3_ on AP surface and COF catalysts were evaluated. The NH_3_ adsorption energies for Tp‐CBH‐Cu‐ClO_4_ and Tp‐CBH‐Cu‐N(NO_2_)_2_ are ‐0.47 and ‐0.59 eV, respectively, much higher than that of AP (‐0.17 eV) and Tp‐CBH (‐0.13 eV), attributing to that the Cu^2+^ sites can accept electrons from NH_3_ (Figure S27, Supporting Information). Therefore, NH_3_ preferred adsorbing on Tp‐CBH‐Cu‐ClO_4_ and Tp‐CBH‐Cu‐N(NO_2_)_2_, which efficiently suppressed NH_3_ accumulation on AP surfaces and eliminated the deactivation period typically observed on LTD stages.

The production of active oxygen species (*O_2_) is identified as a pivotal step in promoting the decomposition of AP. We subsequently evaluated the dissociation energy of HClO_4_ through the cleavage of the Cl‐O bond, which could convert the reactants to active oxygen species.^[^ ^46^ ^]^ In comparison with Tp‐CBH (0.87 eV), Tp‐CBH‐Cu‐ClO_4_ (0.53 eV), and Tp‐CBH‐Cu‐N(NO_2_)_2_ (0.50 eV) show a much lower energy barrier to initiate the dissociation of HClO_4_, making it easier to generate *O_2_ (Figure 5b). Upon adsorption of HClO₄ on the catalyst, the charge density difference analysis for Tp‐CBH, Tp‐CBH‐Cu‐N(NO_2_)_2,_ and Tp‐CBH‐Cu‐ClO_4_ were investigated (Figure 5c). Compared to the Tp‐CBH, the anion in Tp‐CBH‐Cu‐ClO_4_ and Tp‐CBH‐Cu‐N(NO_2_)_2_ further modulates the electronic structure of Cu, influencing the O electron density of HClO_4_ adsorbed on Cu sites. Specifically, the O electron density becoming more concentrated in Tp‐CBH‐Cu‐N(NO_2_)_2_, primarily due to the strong electron‐withdrawing effect of the nitro groups, which creates a more polarized electronic environment that ultimately facilitates easier Cl─O bond dissociation. Therefore, the synergistic effect between the ‐N(NO_2_)_2_ and Cu^2+^ center in Tp‐CBH‐Cu‐N(NO_2_)_2_ promoted the rapid formation of reactive oxygen species, enabling the chemical transformation of NH_3_. This is in good accordance with our experimental findings, that is Tp‐CBH‐Cu‐N(NO_2_)_2_ catalyst rapidly converts AP into NO products, accompanied by concentrated energy release.

Based on real‐time TG‐FTIR‐GC/MS analysis and DFT calculations, we speculated that the thermal decomposition process of AP catalyzed by COF catalytic promoter is as follows: AP first accumulates on the Cu‐decorated COF catalysts and enhances their thermal stability. At the same time, due to the atomically dispersed catalytic sites in the porous framework combined with the abundant energetic anionic groups, Tp‐CBH‐Cu‐N(NO_2_)_2_ and Tp‐CBH‐Cu‐ClO_4_ exhibited a fast AP degradation process companied with massive release of heat. The produced NH_3_ immediately preferred to adsorb on the surface of Cu anchored COF promoters instead of AP surface, and the dissociation ability of intermediate product HClO_4_ to generate active oxygen species significantly increased (Figure 5d). Consequently, the oxidation of NH_3_ into N_2_O, NO, and NO_2_ occurred at extremely narrow temperature range and low temperature, accelerating the AP decomposition rate.

## 3 Conclusion

In summary, we have shown that the usage of single Cu atoms anchored COFs as catalytic promoters could significantly modify the AP's decomposition behavior. Our achievements encompass rapid decomposition kinetics, decreased decomposition temperature, concentrated energy release process, and increased total energy release amount, surpassing previously reported Cu‐based catalysts. Through a combination of comprehensive characterizations and theoretical calculations, the intriguing AP decomposition process is attributed to maximized exposure of active sites, and the engineering of microenvironment of single‐form Cu sites via anionic groups facilitated the breakdown of Cl‐O bond of the decomposition intermediate HClO_4_ and increased the oxidation ability of NH_3_ to nitrogen‐containing oxides. This work provides new insights for the design and synthesis of efficient catalytic promoters for high‐performance propellant applications.

## Conflict of Interest

The authors declare no conflict of interest.

## Supporting information



Supporting Information

## Data Availability

The data that support the findings of this study are available in the supplementary material of this article.
