# Supplementary material for: Single Cu Atoms Anchored Energetic COFs as Combustion Catalytic Promoters toward Rapid and Concentrated Thermal Decomposition of Ammonium Perchlorate
Source: Adv Sci (Weinh). 2025 May 14;12(29):e01761. doi: 10.1002/advs.202501761 (PMC12362789; doi:10.1002/advs.202501761)
Supplement: Supplementary file 1 — Supporting Information [file ADVS-12-e01761-s001.docx]

Single Cu Atoms Anchored Energetic COFs as Combustion Catalytic Promoters toward Rapid and Concentrated Thermal Decomposition of Ammonium Perchlorate

Meng Zhang,^1,2§^ Shan Wang,^3§^ Xu-Yang Wang,^4^ Zhi-Peng Wu,^4^ Bai-Suo Ding,^3,6^ Li Yang,^4^ Wen-Chao Tong,^2,4^ Qinglang Ma,^1,5,6^ and Qian-You Wang^3,5^*

*^1^* Frontiers Science Center for High Energy Material, Advanced Research Institute of Multidisciplinary Science, Beijing Institute of Technology, Beijing 100081, China

*^2^* Science and Technology on Applied Physical Chemistry Laboratory, Shaanxi Applied Physics-Chemistry Research Institute, Xi’an, Shaanxi 710061, China

*^3^* School of Chemistry and Chemical Engineering, Beijing Institute of Technology, Beijing 100081, China

*^4^* Key Laboratory of Explosion Science and Technology of China, Beijing Institute of Technology, Beijing 100081, China

*^5^* Zhengzhou Academy of Intelligent Technology; Beijing Institute of Technology; Zhengzhou 450000, China.

*^6^* Chongqing Advanced Materials Institute; Chongqing 408107; China

Part 1. Experimental section

1. Materials and Reagents.

All chemicals and solvents used were obtained from suppliers without further purification.

1. Measurements

Powder X-ray diffraction (PXRD) was collected on a Riguku D / Max-2500PC X-ray diffractometer with Cu sealed tub (λ = 1.54178 Å).

Fourier transform infrared (FT-IR) spectra was recorded on a Bruker ALPHA II FT-IR spectrometer.

The X-ray photoelectron spectroscopy (XPS) measurements were tested using Thermo Scientific K-Alpha. The C peak at 284.8 eV was used as a reference to correct for charging effects.

Nitrogen sorption isotherms were measured at liquid nitrogen temperature (77 K) by using automatic volumetric adsorption equipment (Belsorp Max) after a degassed process at 120 ℃ for 12 h. Specific surface areas were obtained by using the Brunauer-Emmet-Teller (BET) model, pore size distributions were simulated by the nonlocal density functional theory (NLDFT) model.

The inductively coupled plasma mass spectrometry (ICP-MS) analysis was recorded on Agilent 5110 (OES).

Morphology of all samples were carried out using Zeiss Sigma 500 on a scanning electron microscopy (SEM) measurement.

Transmission electron microscopy (TEM) images were obtained in FEI TalosF200S. Atomic-resolution high-angle annular dark-field scanning transmission electron microscopy (HAADF-STEM) images were obtained in FEI Titan cubed Themis G2 300 STEM with aspherical aberration corrector.

Thermogravimetric analysis (TGA) was recorded by a METTLER TGA 2 SF/1100 thermal analyzer from room temperature to 800 ℃ under N_2_ atmosphere using a heating rate of 10 ℃/min.

Differential scanning calorimetry (DSC) was performed on a METTLER DSC 3 500/Fan with corresponding heating rates from 30 to 500 °C. The test sample was placed in platinum sample pans and analyzed using a dry nitrogen atmosphere with a flow rate of 20 mL·min^-^¹.

TG-FTIR-GC/MS experiment was performed on PerkinElmer TGA8000-Frontier-Clarus590/SQ8S with the heating rate of 10 °C·min^−1^ in helium atmosphere. A 5 mg sample was placed on alumina crucible and the temperature was ramped from 50 °C to 500 °C.

1. XANES supporting information.

Data reduction, data analysis, and EXAFS fitting were performed and analyzed with the Athena and Artemis programs of the Demeter data analysis packages^1^ that utilizes the FEFF6 program^2^ to fit the EXAFS data. The energy calibration of the sample was conducted through standard and Cu foil, which as a reference was simultaneously measured. A linear function was subtracted from the pre-edge region, then the edge jump was normalized using Athena software. The χ(k) data were isolated by subtracting a smooth, third-order polynomial approximating the absorption background of an isolated atom. The k3-weighted χ(k) data were Fourier transformed after applying a HanFeng window function (Δk = 1.0). For EXAFS modeling, the global amplitude EXAFS (CN, R, σ2 and ΔE_0_) were obtained by nonlinear fitting, with least-squares refinement, of the EXAFS equation to the Fourier-transformed data in R-space, using Artemis software, EXAFS of the Cu foil are fitted and the obtained amplitude reduction factor S02 value (0.802) was set in the EXAFS analysis to determine the coordination numbers (CNs) in sample.

1. Computational methods.

All the calculations were performed within the framework of the density functional theory (DFT) as implemented in the Vienna Ab initio Software Package (VASP 5.4.4) code within the Perdew-Burke-Ernzerhof (PBE) generalized gradient approximation and the projected augmented wave (PAW) method.^3^ 1-4 The cutoff energy for the plane-wave basis set was set to 450 eV. The Brillouin zone of the surface unit cell was sampled by Monkhorst-Pack (MP) grids, with k-point mesh density of 2π × 0.04 Å^-1^ for structures optimizations. The convergence criterion for the electronic self-consistent iteration and force was set to 10^-5^ eV and 0.01 eV/Å, respectively. The vacuum layer of 15 Å was introduced to avoid interactions between periodic images. The free energies of adsorbates at temperature T were estimated according to the harmonic approximation, and the entropy is evaluated using the following equation:

$$\sum_{i}^{harm DOF} \left( \frac{\varepsilon_{i}}{K_{B}T(e^{\frac{\varepsilon_{i}}{K_{B}T}}-1)}-\ln(1-e^{-\frac{\varepsilon_{i}}{K_{B}T}}) \right)$$

where KB is Boltzmann’s constant and DOF is the number of harmonic energies (εi) used in the summation denoted as the degree of freedom, which is generally 3N, where N is the number of atoms in the adsorbates. Meanwhile, the free energies of gas phase species are corrected as:

$$G_{g}(T)=E_{elec}+E_{ZPE}+\int C_{p}dT-TS(T)$$

where Cp is the gas phase heat capacity as a function of temperature derived from Shomate equations and the corresponding parameters in the equations were obtained from NIST.

5. Catalytic Performance in the Thermal Decomposition of AP

The prepared catalysts were mixed with AP (with a mass ratio of catalysts to the mixed sample of 10%) in a mortar and ground lightly using MeOH until well mixed. The prepared mixture was used for DSC measurement with 5, 10, 15, 20 °C min^−1^ from 50 to 500 °C under N_2_ atmosphere in aluminum crucible. For each test, the amount of the mixture used in each test was controlled within 0.5 to 1 mg.

1. Synthesis of Tp-CBH.


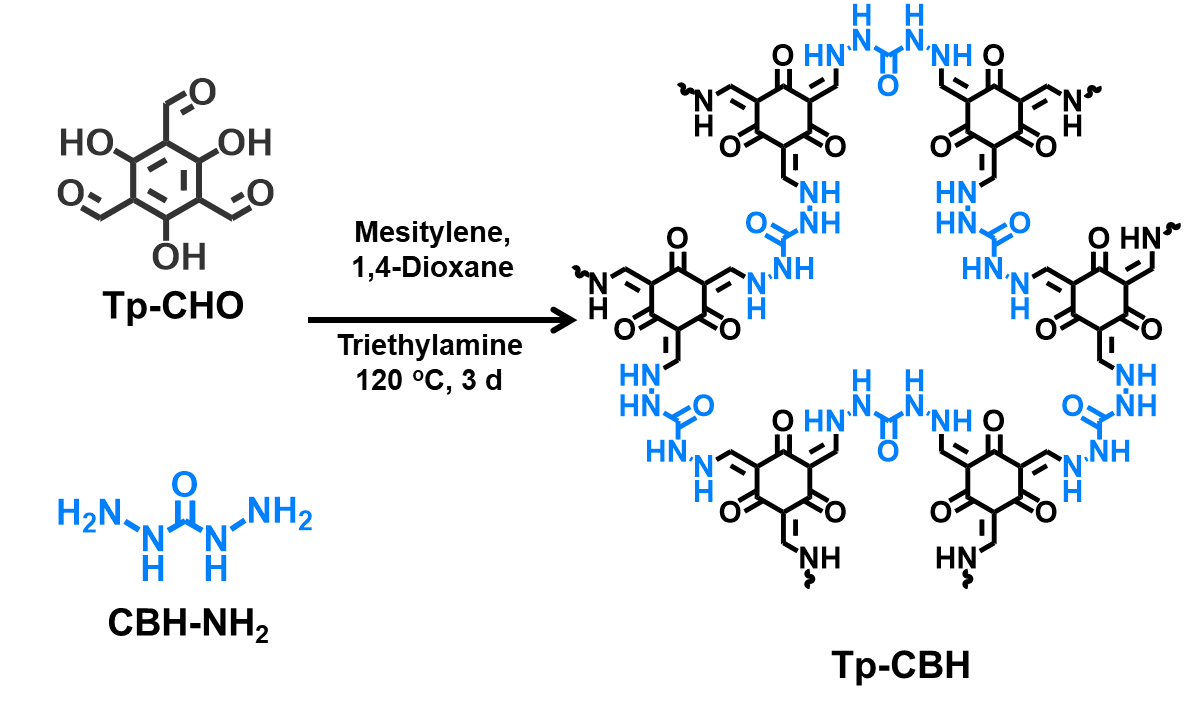


Figure S1. Schematic of the synthesis of Tp-CBH.

A Pyrex tube was charged with Tp (21 mg，0.1 mmol), CBH (13.5 mg，0.15 mmol), 1.3 mL 1,4-dioxane, and 0.7 mL 1,3,5-trimethylbenzene. After sonication for about 15 minutes to disperse evenly, 40 μL triethylamine was added. The tube was flash-frozen at 77 K (liquid N_2_ bath) and degassed through three freeze-pump-thaw cycles by evacuated through an oil pump and then sealed under vacuum. After warming to room temperature, the mixture was heated at 120 ºC for 72 hours. A dark orange precipitate was isolated by vacuum filtration, then washed with THF until the filtrate was colorless. The powder sample was then transferred to a Soxhlet extractor and washed with THF for 24 h.

1. Synthesis of Tp-CBH-Cu-ClO_4_.

Tp-CBH (14.55 mg, 0.15 mmol) and Cu(ClO_4_)_2_·6H_2_O (167 mg, 0.45 mmol) were dispersed in 10 mL EtOH, and stirred at room temperature for 12 hours. The solid was collected by vacuum filtration, then washed with EtOH until the filtrate was colorless. The mixture was vacuum-dried at 60 °C for 12 h to afford Tp-CBH-Cu-ClO_4_ as orange powder.

1. Synthesis of Tp-CBH-Cu-N(NO_2_)_2_.

Tp-CBH (14.55 mg, 0.15 mmol) and Cu[N(NO_2_)_2_]_2_ (148.5 mg, 0.45 mmol) were dispersed in 10 mL EtOH, and stirred at room temperature for 12 hours. The solid was collected by vacuum filtration, then washed with EtOH until the filtrate was colorless. The mixture was vacuum-dried at 60 °C for 12 h to afford Tp-CBH- Cu-N(NO_2_)_2_ as orange powder.

Table S1. The EA analysis in Tp-CBH, Tp-CBH-Cu-ClO_4_ and Tp-CBH-Cu-N(NO_2_)_2._

| Sample | C% | | H% | | N% | |
| --- | --- | --- | --- | --- | --- | --- |
|  | Anal. | Exp. | Anal. | Exp. | Anal. | Exp. |
| Tp-CBH | 43.30 | 43.04 | 4.48 | 4.27 | 28.88 | 22.87 |
| Tp-CBH-Cu-ClO_4_ | 35.29 | 34.48 | 2.52 | 2.55 | 23.52 | 20.34 |
| Tp-CBH-Cu-N(NO_2_)_2_ | 35.03 | 34.09 | 2.50 | 2.45 | 23.3 | 20.96 |

Table S2. The Cu contents in Tp-CBH-Cu-ClO_4_ and Tp-CBH-Cu-N(NO_2_)_2._

| Sample | Cu content |
| --- | --- |
| Tp-CBH-Cu-ClO_4_ | 6.75 wt% |
| Tp-CBH-Cu-N(NO_2_)_2_ | 5.2 wt% |

Part 2. Supplementary Figures and Tables


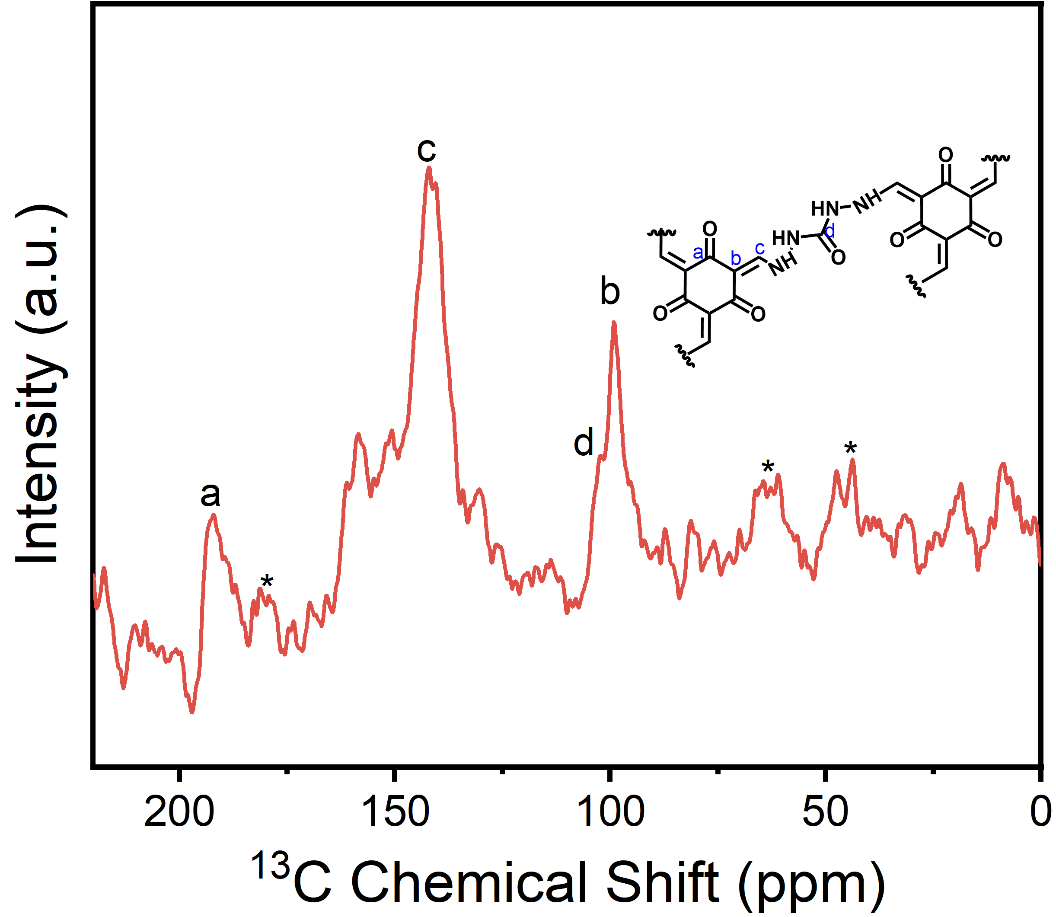


Figure S2. Solid-state ^13^C CP/MAS NMR spectrum of Tp-CBH.


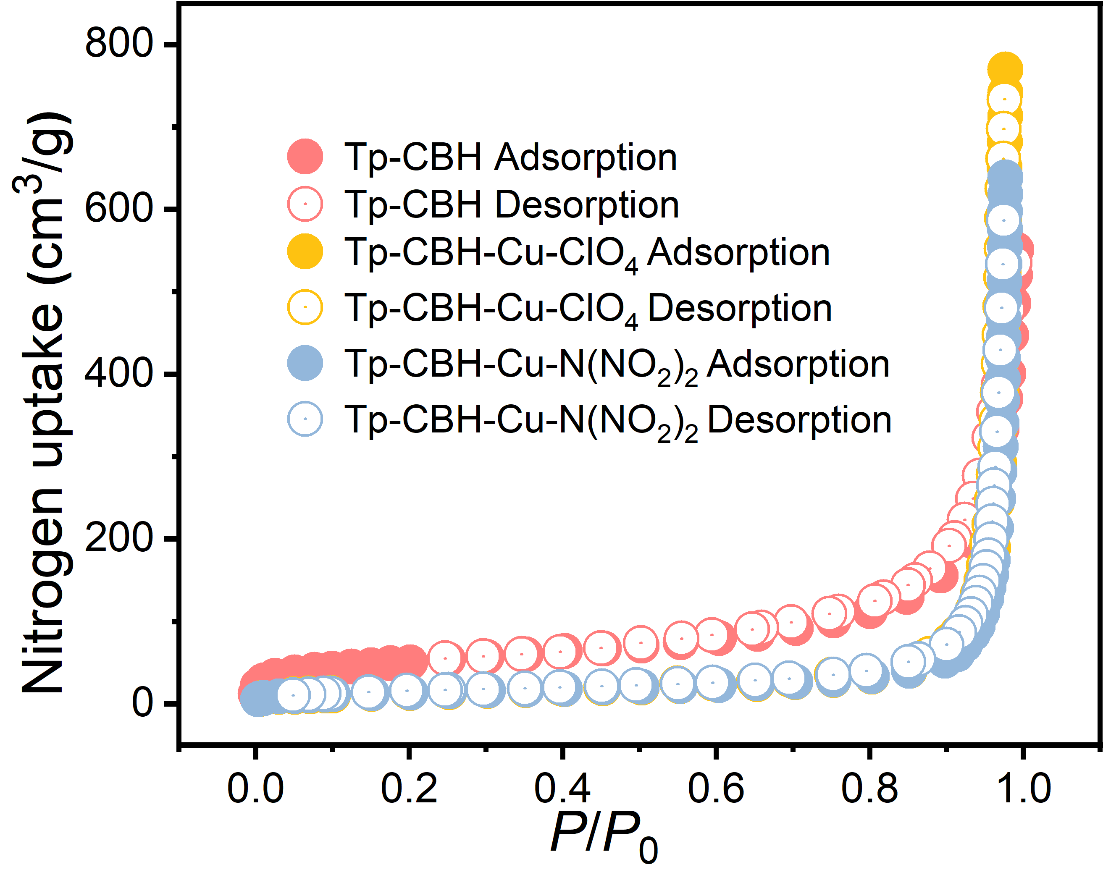


Figure S3. N_2_ sorption isotherms of Tp-CBH, Tp-CBH-Cu-ClO_4_, and Tp-CBH-Cu-N(NO_2_)_2_.


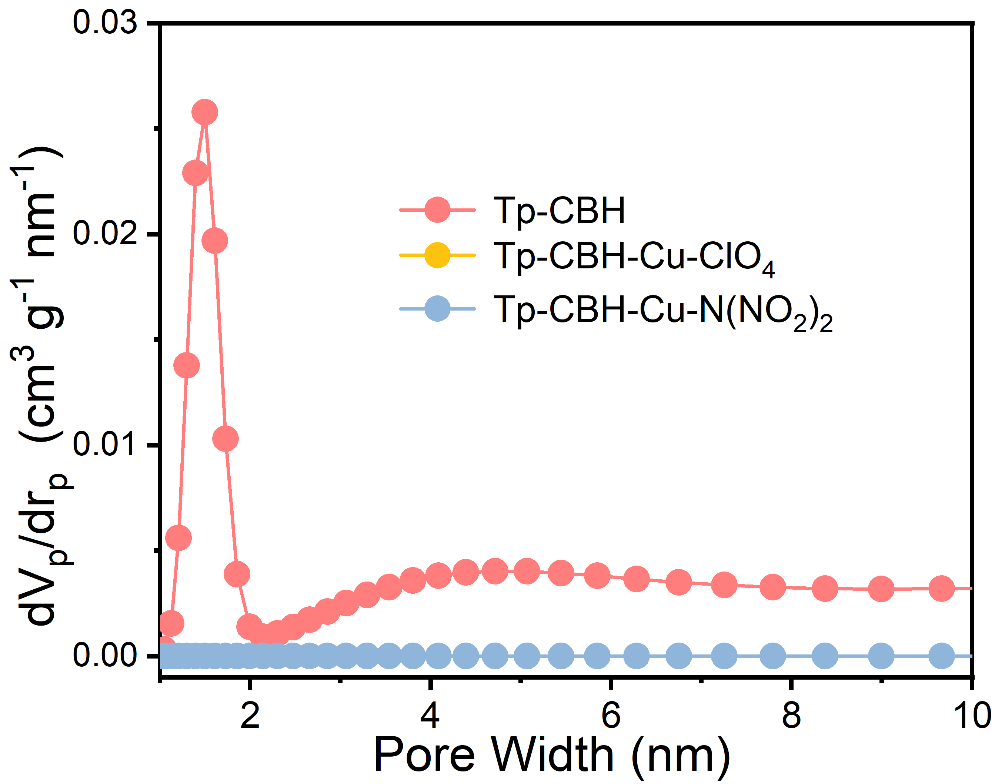


Figure S4. Pore-size distribution profiles of Tp-CBH, Tp-CBH-Cu-ClO_4_, and Tp-CBH-Cu-N(NO_2_)_2_.


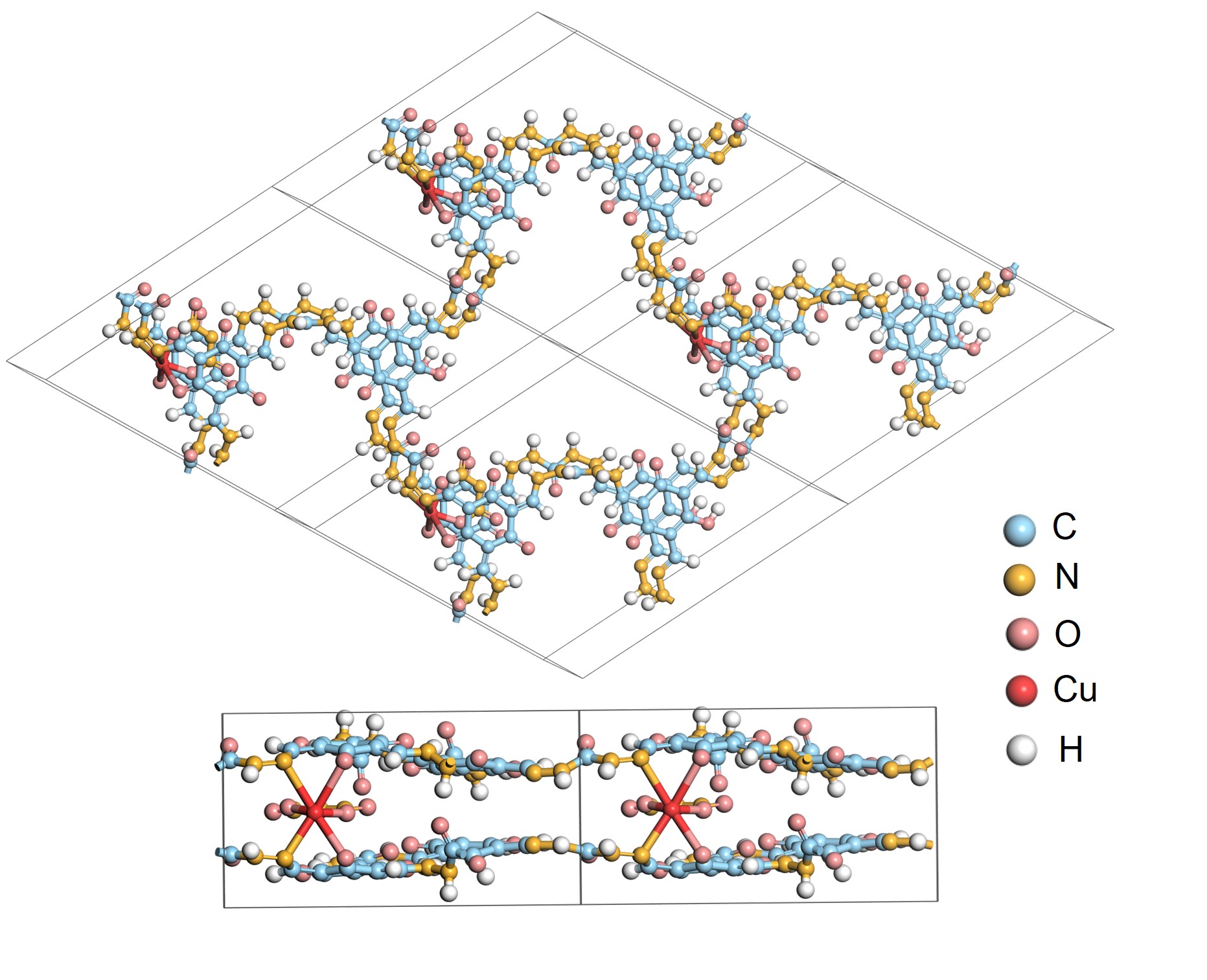


Figure S5. Refined structural models of Tp-CBH-Cu-N(NO_2_)_2_.


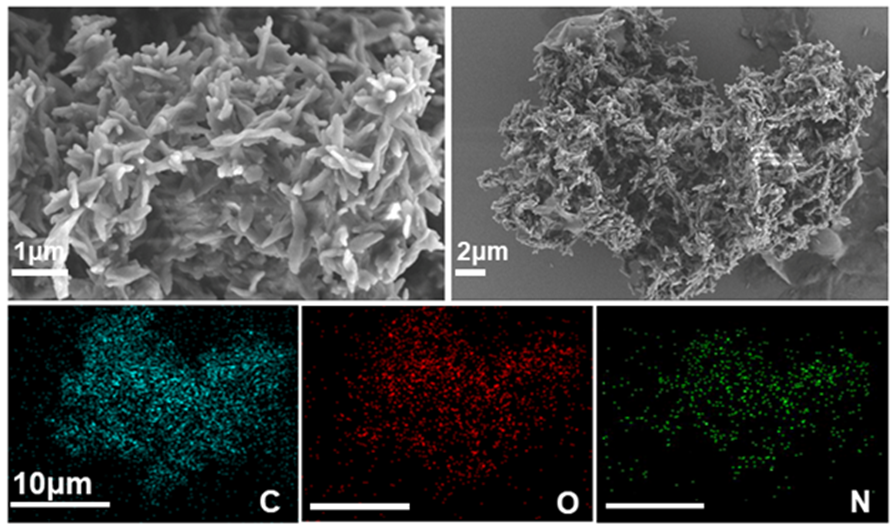


Figure S6. SEM image and corresponding elemental mapping of Tp-CBH.


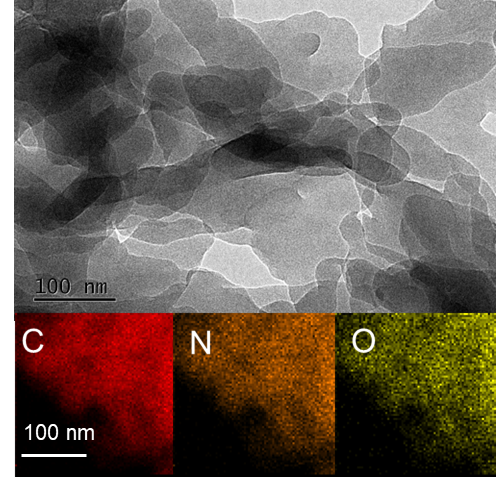


Figure S7. TEM image and corresponding elemental mapping of Tp-CBH.


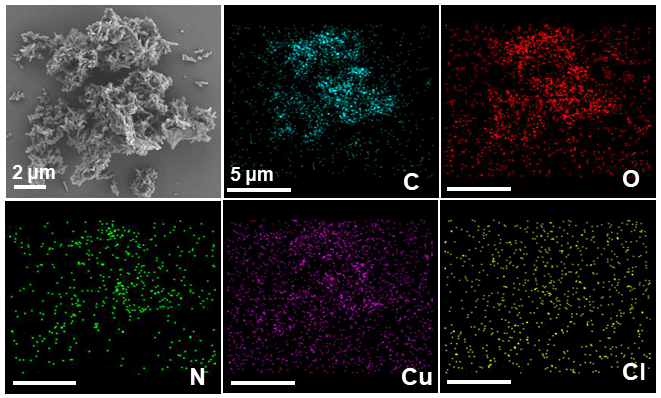


Figure S8. SEM image and corresponding elemental mapping of Tp-CBH-Cu-ClO_4_.


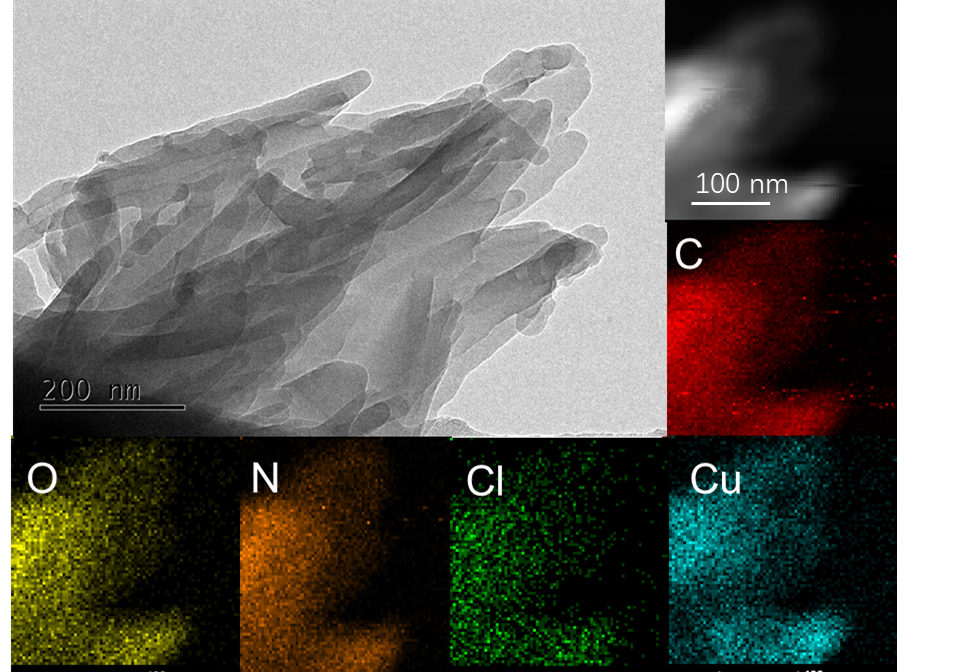


Figure S9. TEM image and corresponding elemental mapping of Tp-CBH-Cu-ClO_4_.


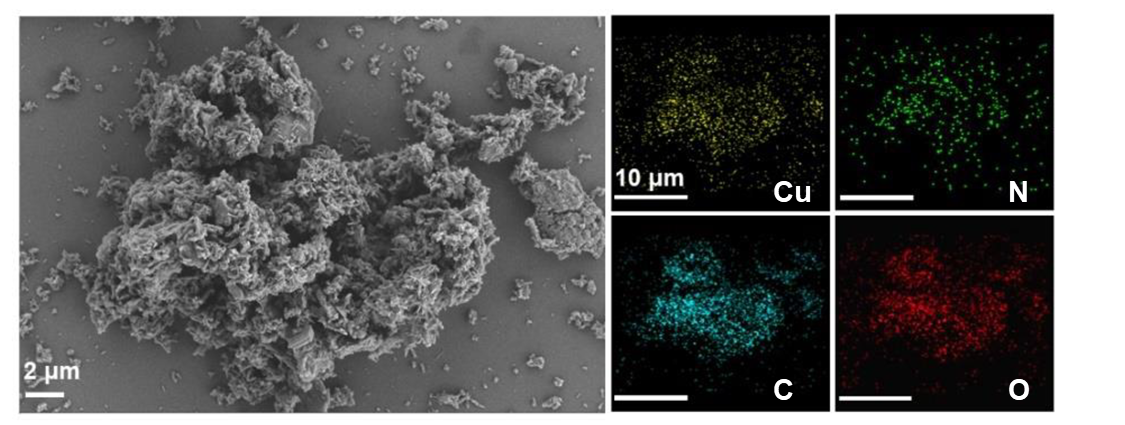


Figure S10. SEM images and corresponding elemental mapping of Tp-CBH-Cu-N(NO_2_)_2_.


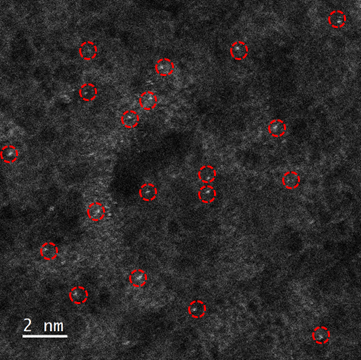


Figure S11. Atomic-resolution HAADF-STEM image of P Tp-CBH-Cu-ClO_4_ revealing the atomically dispersed Cu SA.


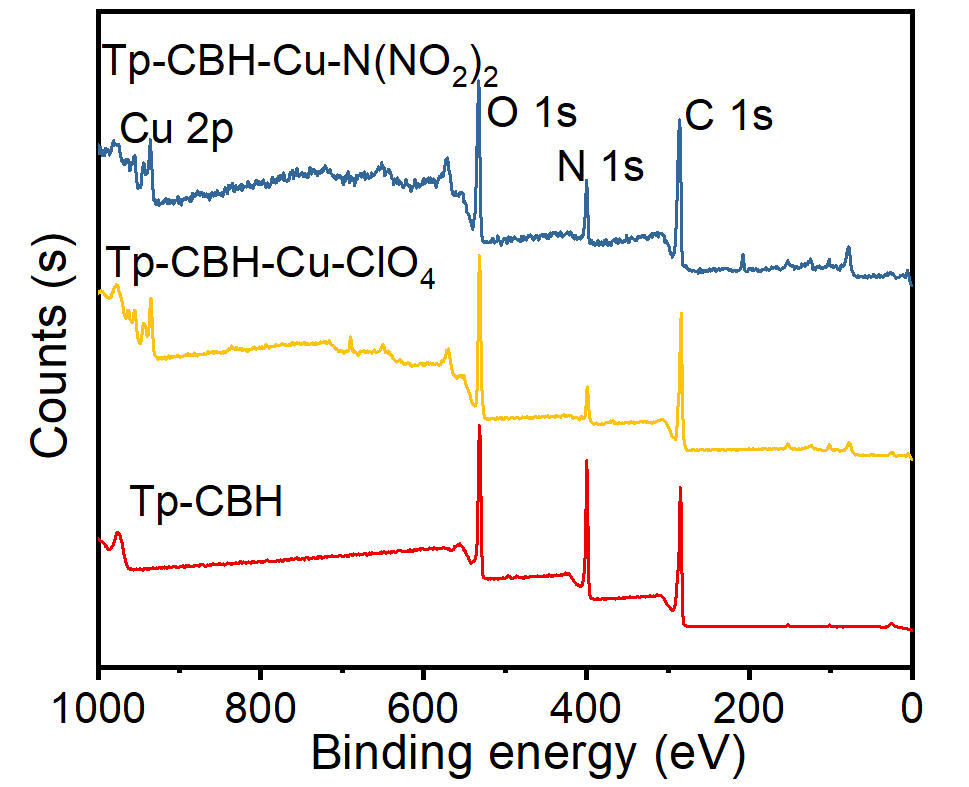


Figure S12. (a) Full XPS spectra of Tp-CBH, Tp-CBH-Cu-ClO_4_, and Tp-CBH-Cu-N(NO_2_)_2_.


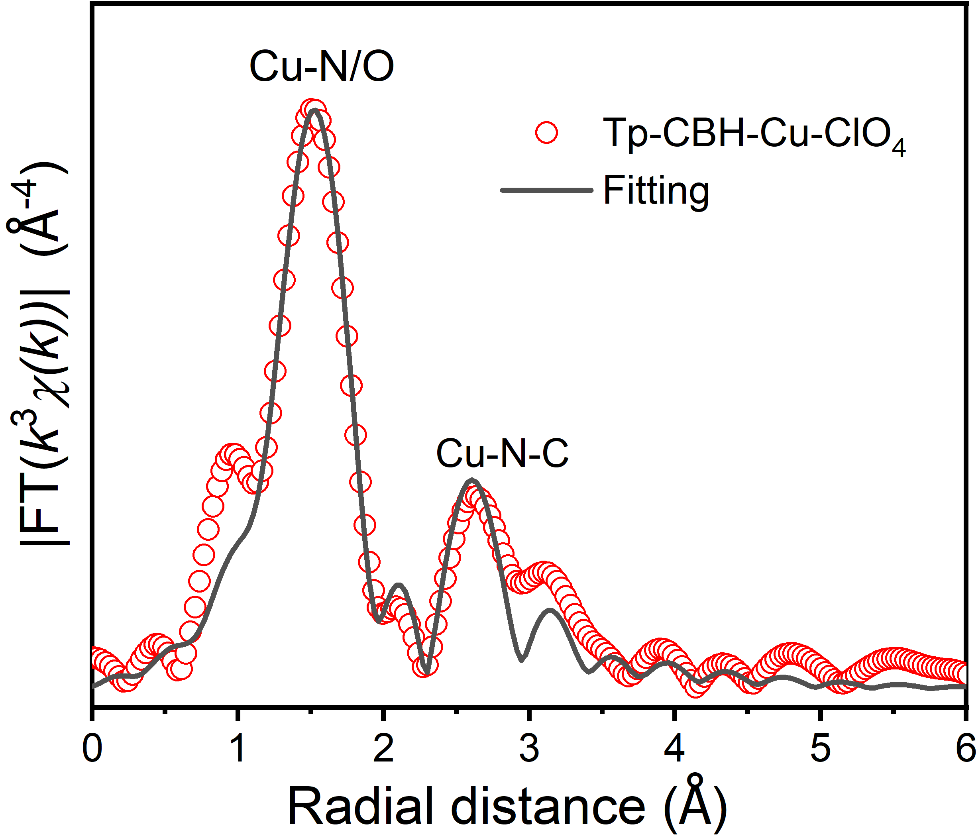


Figure S13. EXAFS R-space fitting curves of Cu species in Tp-CBH-Cu-ClO_4._

_
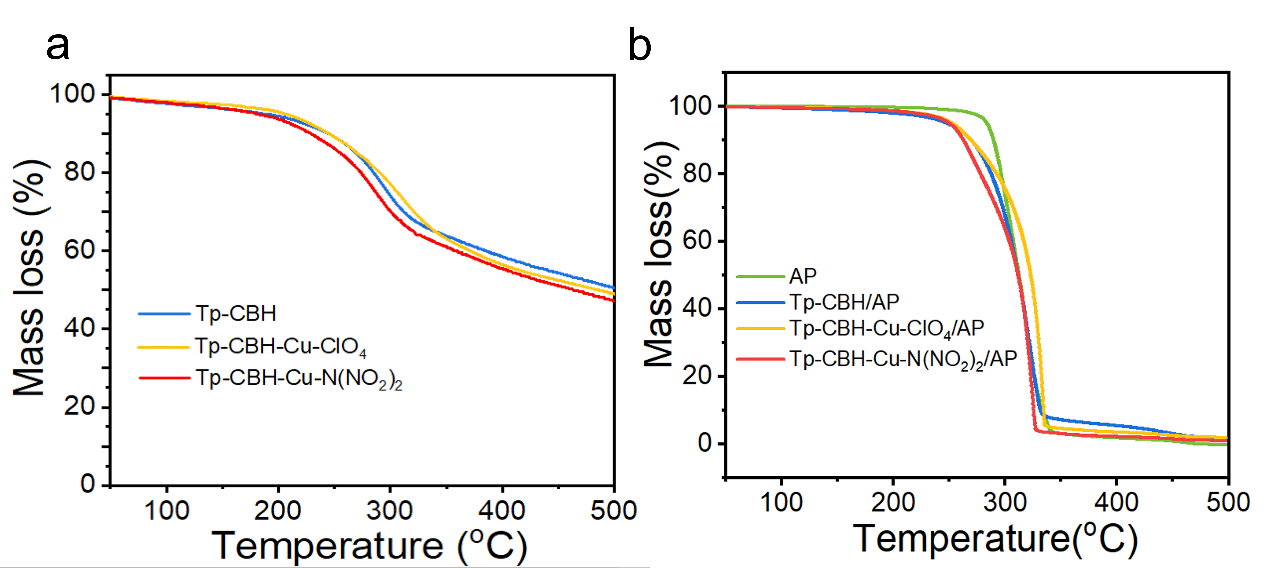
_

Figure S14. TGA profile of (a) Tp-CBH, Tp-CBH-Cu-ClO_4_, Tp-CBH-Cu-N(NO_2_)_2._ (b) Tp-CBH/AP mixture, Tp-CBH-Cu-ClO_4_/ AP mixture, and Tp-CBH-Cu-N(NO_2_)_2_/AP mixture.


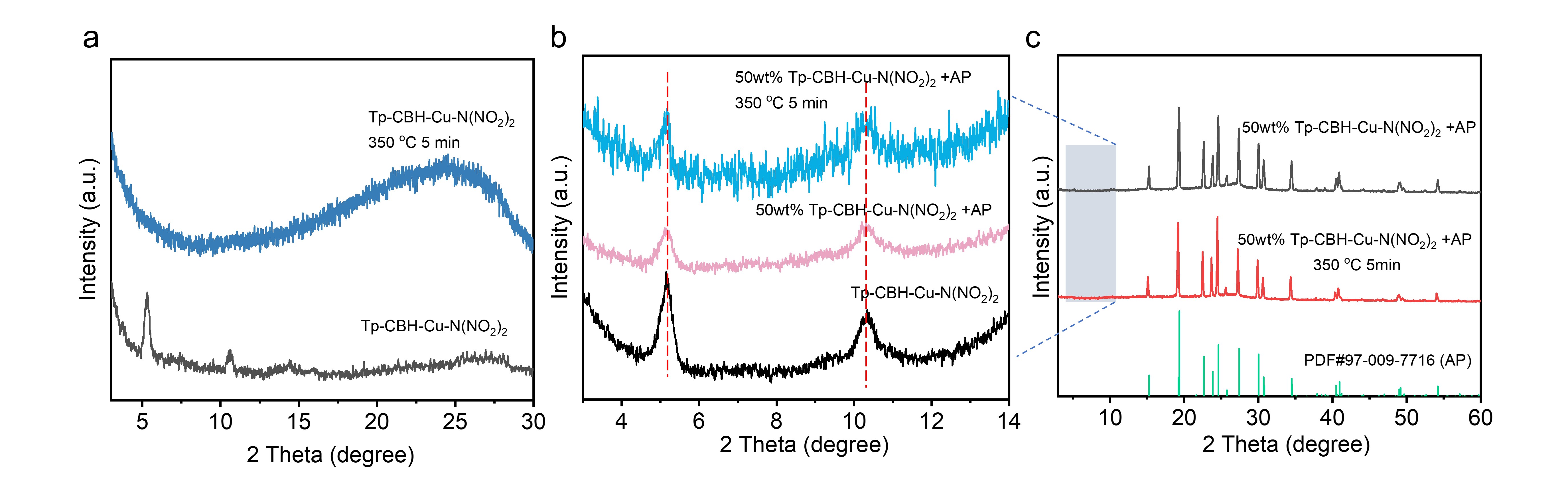


Figure S15. PXRD patterns of (a) pure Tp-CBH-Cu-N(NO_2_)_2_, (b)magnified and (c) full spectrum of 50 wt% Tp-CBH-Cu-N(NO_2_)_2_+AP before and after heating at 350 °C for 5 min.


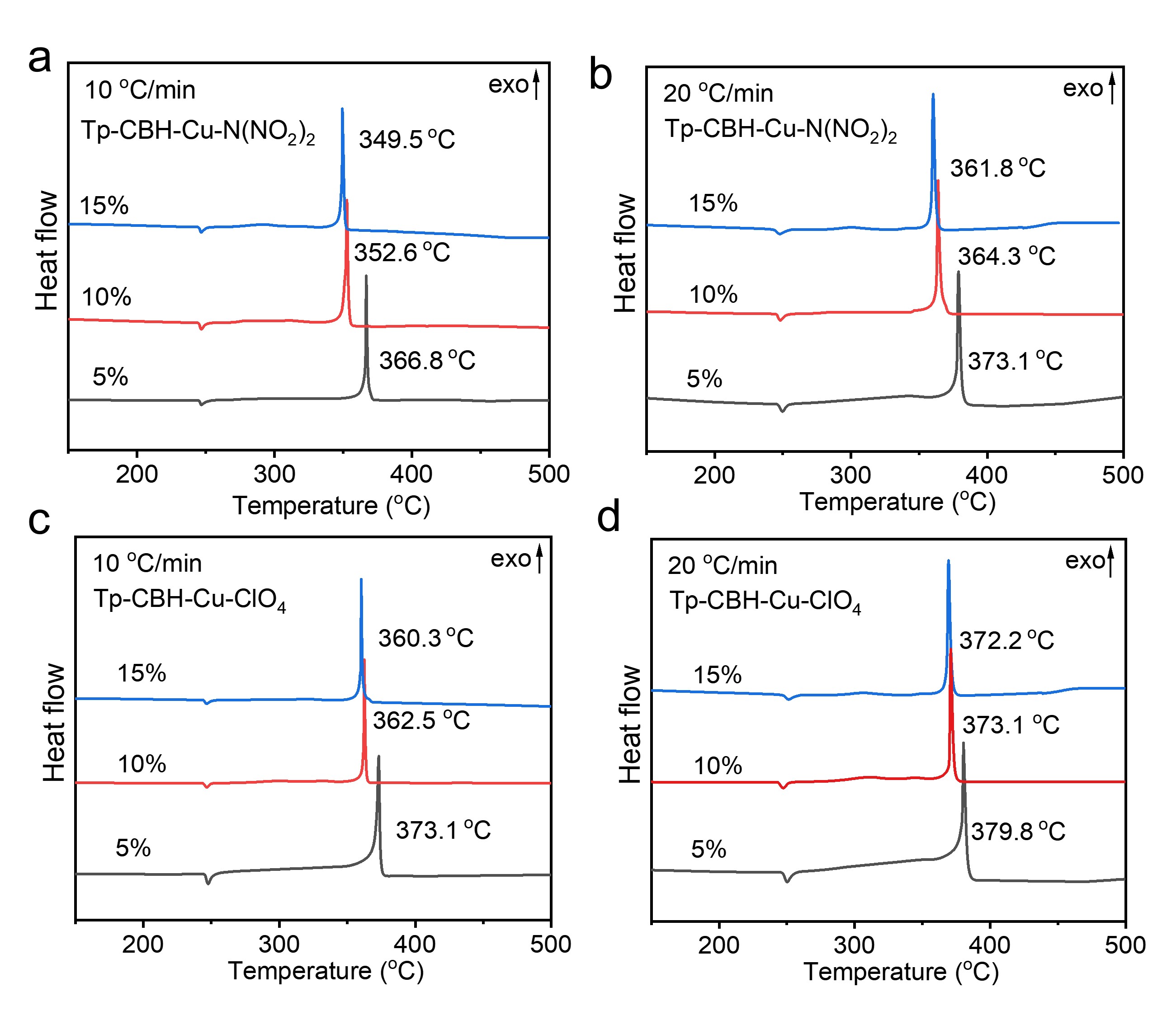


Figure S16. DSC curve of 5%, 10%, and 15% loading of Tp-CBH-Cu-N(NO_2_)_2_ at (a) 10 °C/min and (b) 20 °C/min. DSC curve of 5%, 10%, and 15% loading of Tp-CBH-Cu-ClO_4_ at (c) 10 °C/min and (d) 20 °C/min.


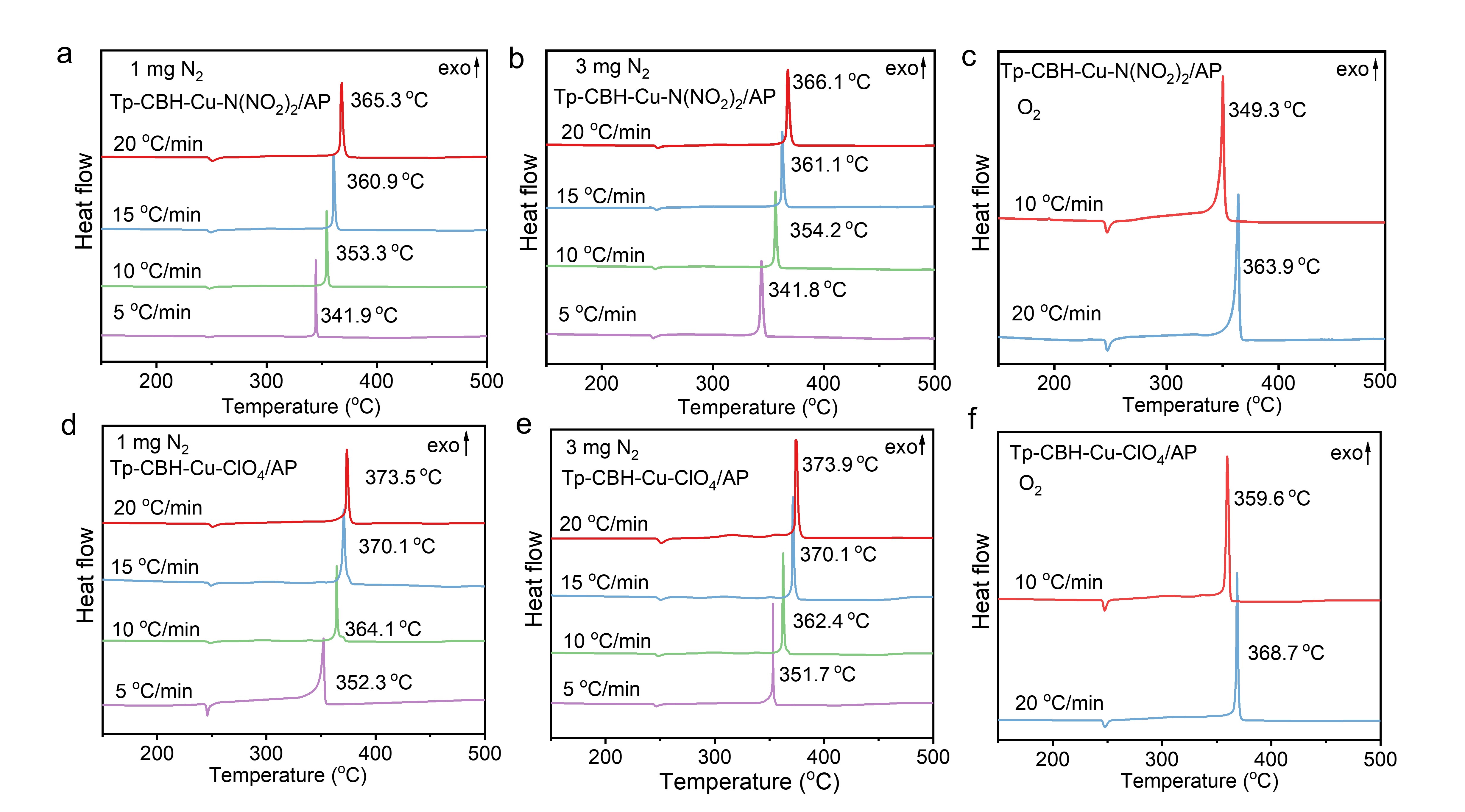


Figure S17. DSC curves of (a) 1 mg Tp-CBH-Cu-N(NO_2_)_2_/AP in N_2_, (b) 3 mg Tp-CBH-Cu-N(NO_2_)_2_/AP in N_2_, (c) Tp-CBH-Cu-N(NO_2_)_2_/AP in O_2_, (d) 1 mg Tp-CBH-Cu-ClO_4_/AP in N_2_, (e) 3 mg Tp-CBH-Cu-ClO_4_/AP in N_2_ (f) Tp-CBH-Cu-ClO_4_/AP in O_2_.


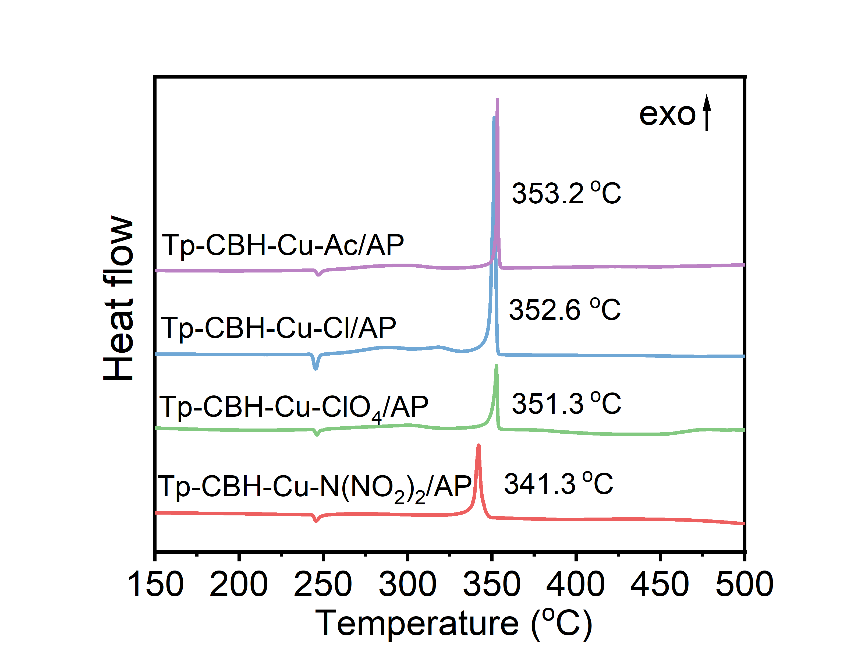


Figure S18. DSC curves for various catalysts/AP mixtures at 5 °C min^-1^.


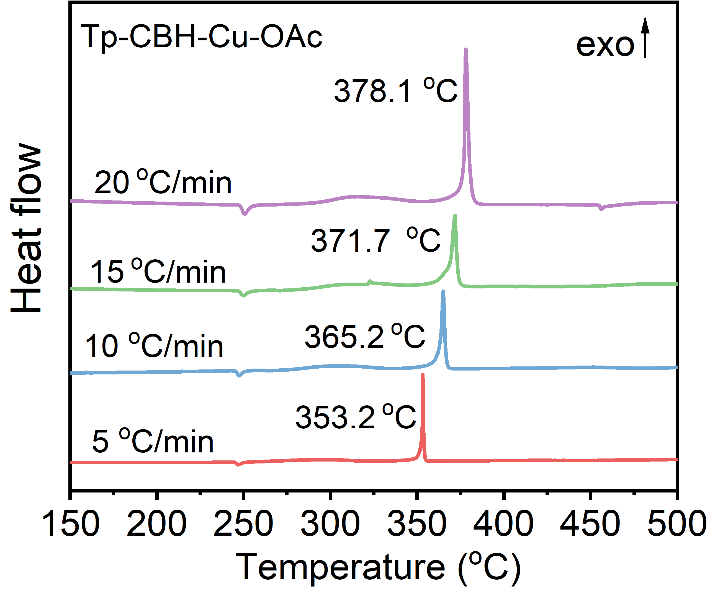


Figure S19. DSC curves of Tp-CBH-Cu-OAc catalyzed thermal decomposition of AP under different heating rates.


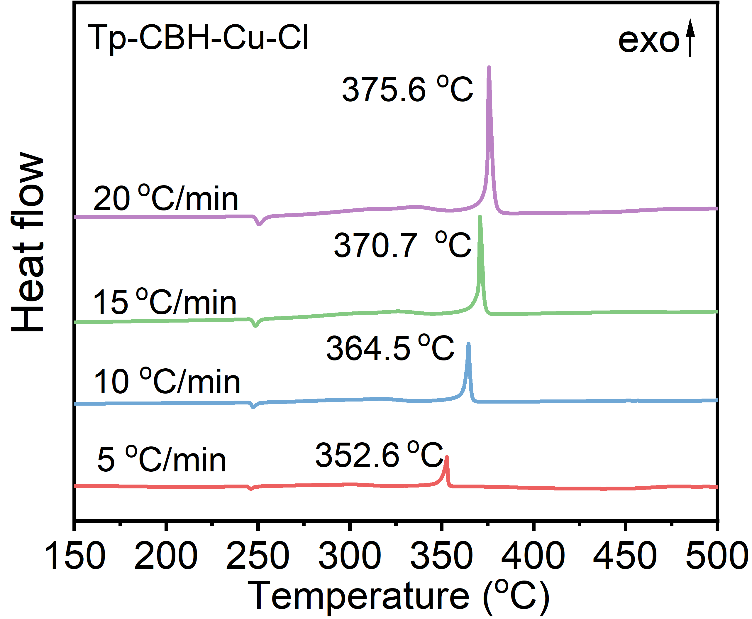


Figure S20. DSC curves of Tp-CBH-Cu-Cl catalyzed thermal decomposition of AP under different heating rates.

Figure S21. DSC curves of Tp-CBH, Tp-CBH-Cu-ClO_4_, and Tp-CBH-Cu-N(NO_2_)_2_ at 5 °C/min.


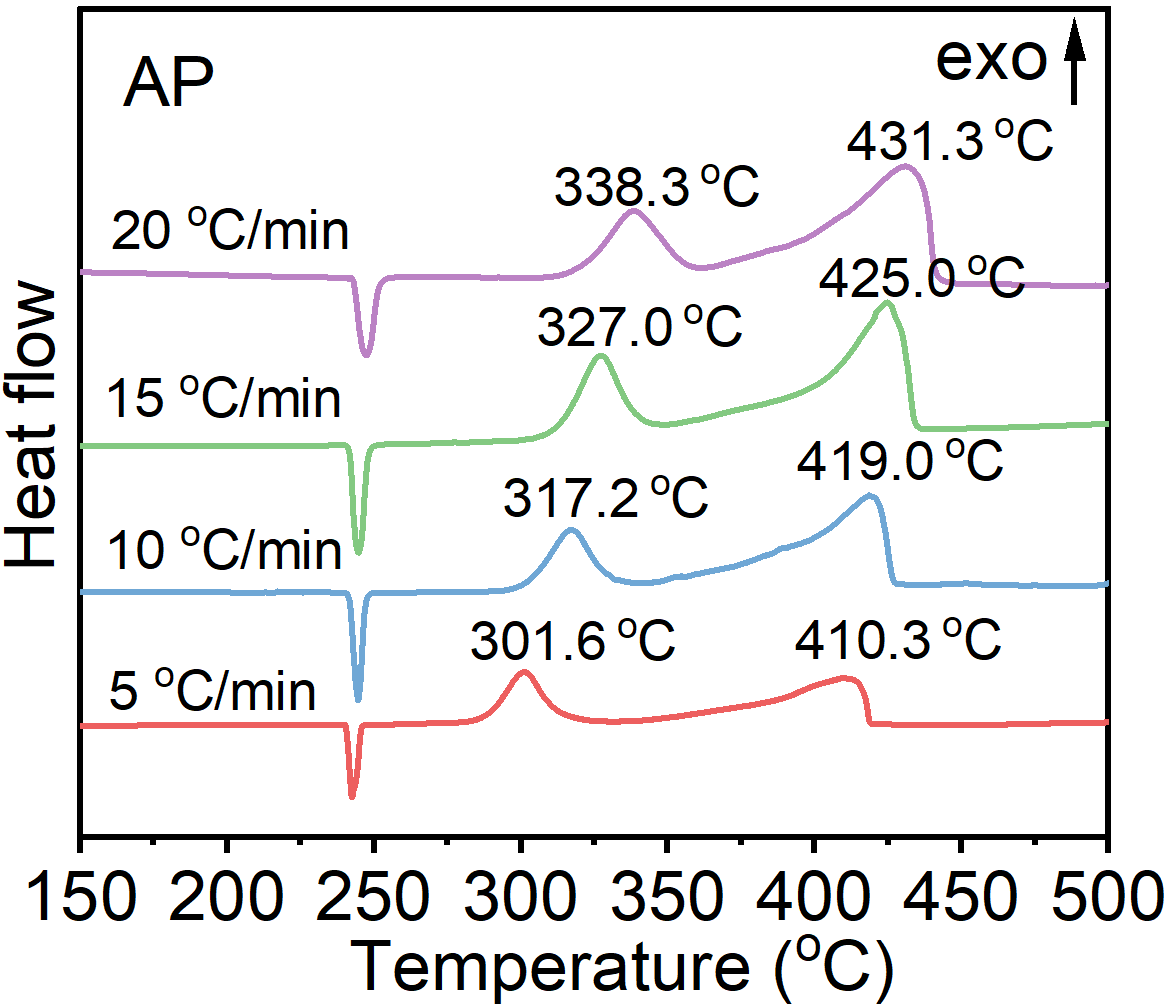


Figure S22. DSC curves of thermal decomposition of AP under different heating rates.


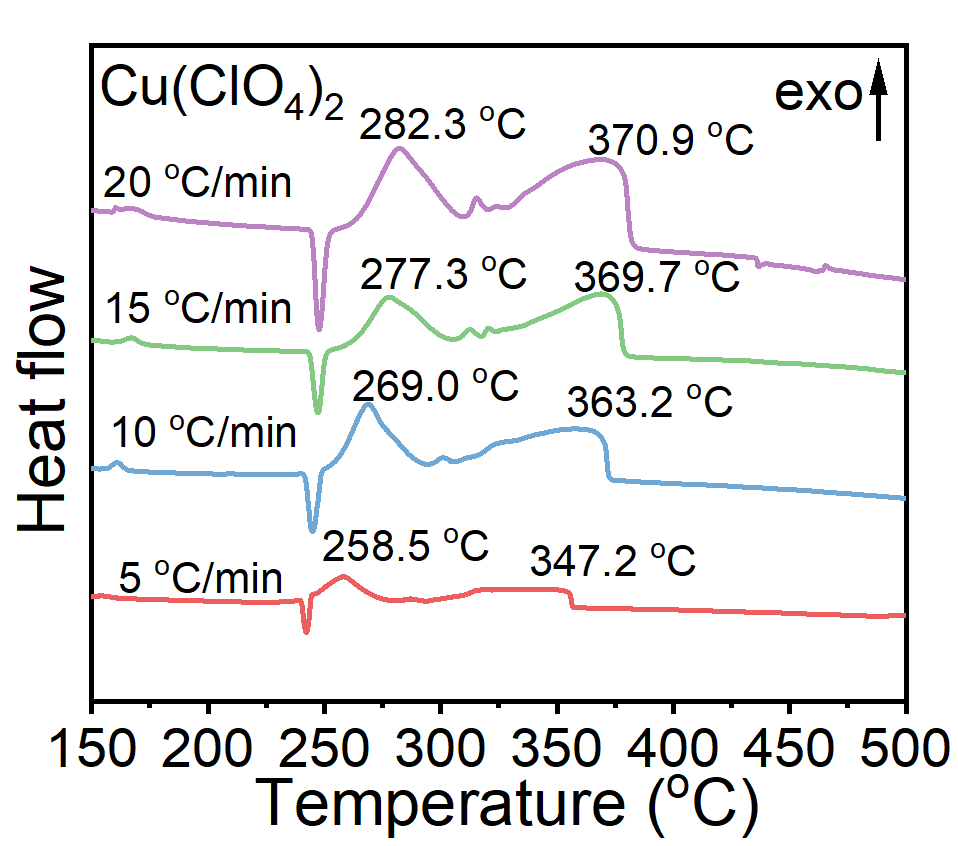


Figure S23. DSC curves of Cu(ClO_4_)_2_ catalyzed thermal decomposition of AP under different heating rates.


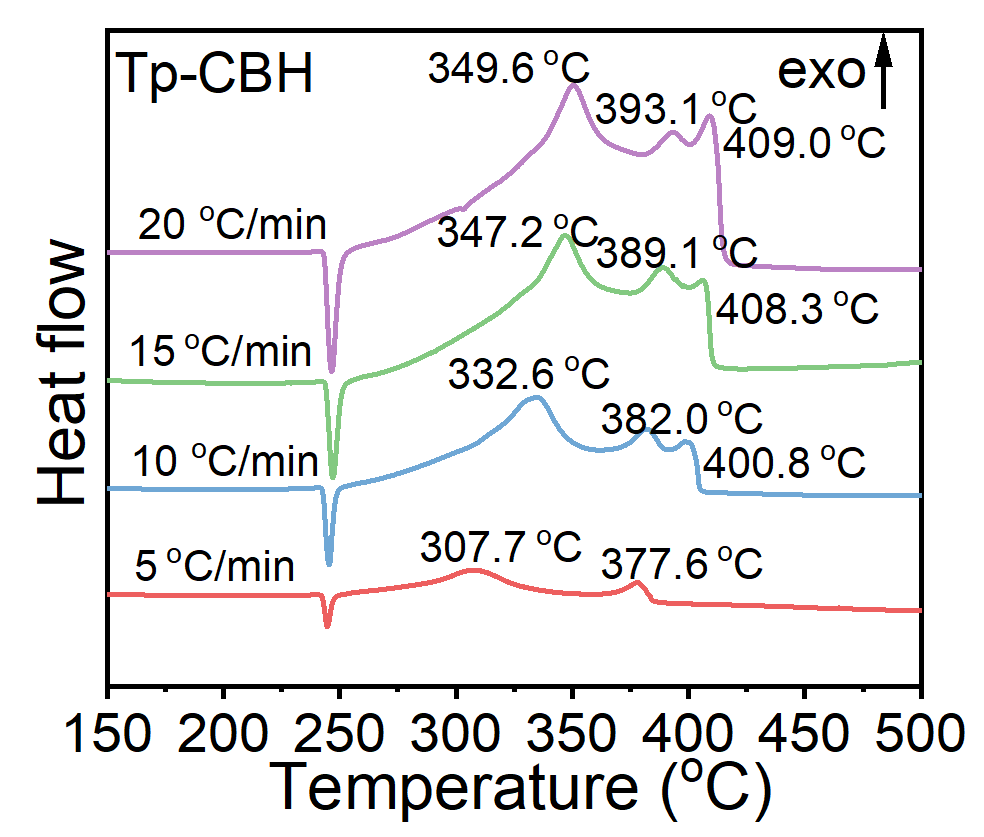


Figure S24. DSC curves of Tp-CBH catalyzed thermal decomposition of AP under different heating rates.


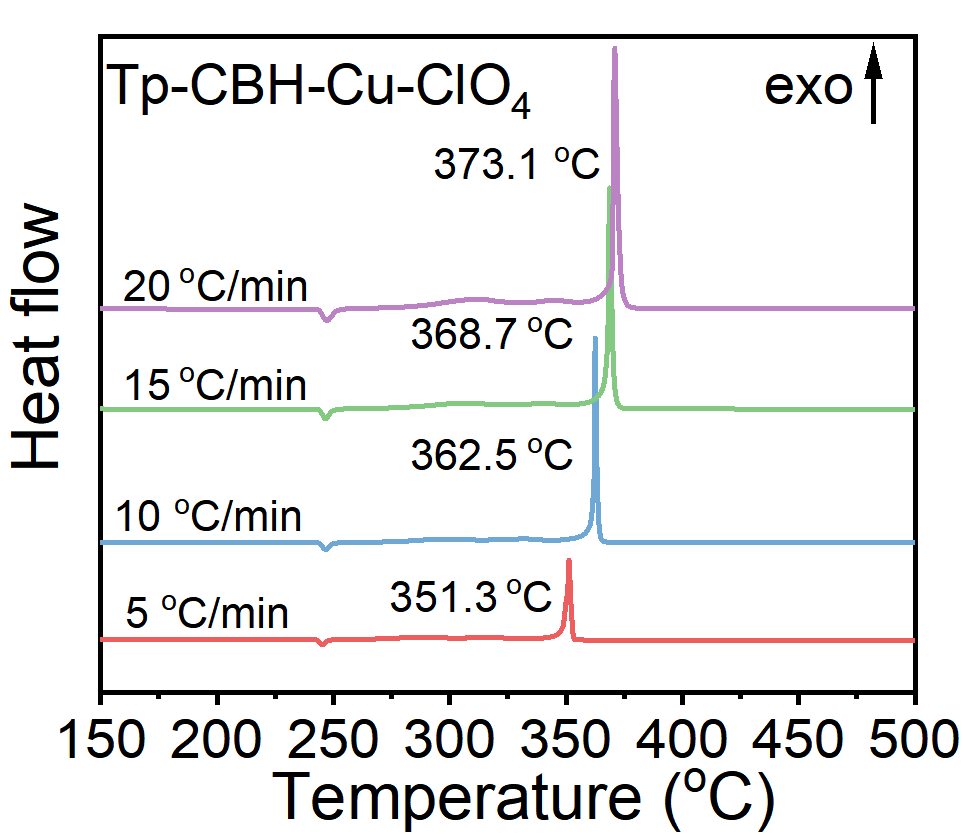


Figure S25. DSC curves of Tp-CBH-Cu-ClO_4_ catalyzed thermal decomposition of AP under different heating rates.


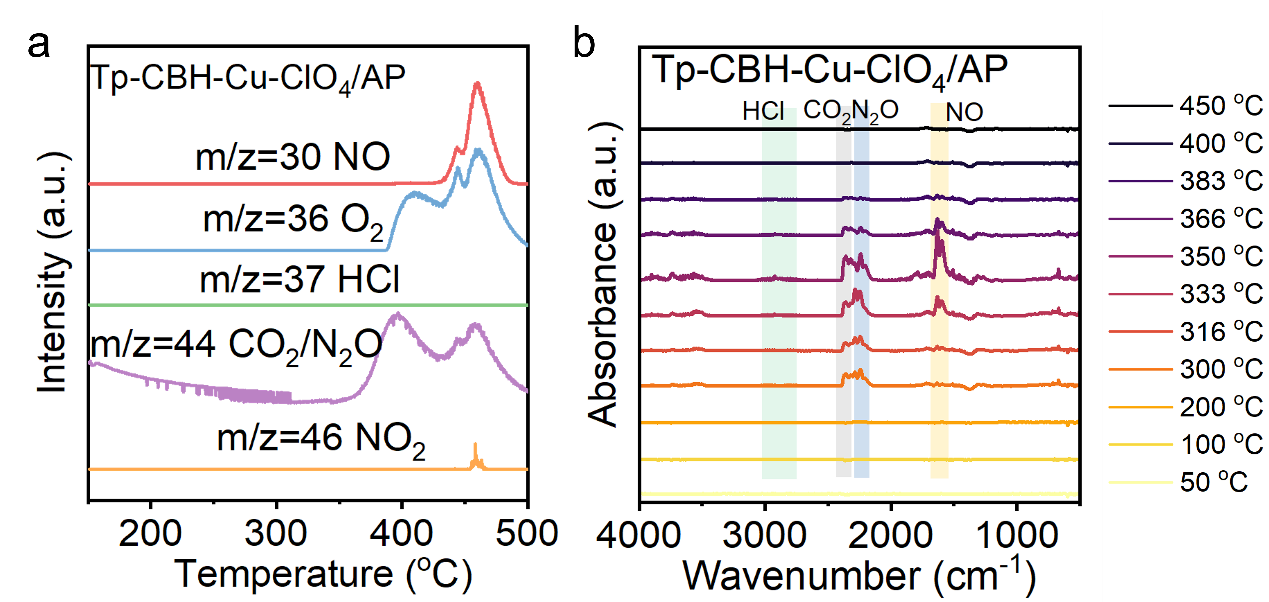


Figure S26. (a) *In situ* TG-MS spectral analysis of gas production in Tp-CBH-Cu-ClO_4_/AP. (b) *In situ* TG-FTIR test results of gas production Tp-CBH-Cu-ClO_4_/AP.


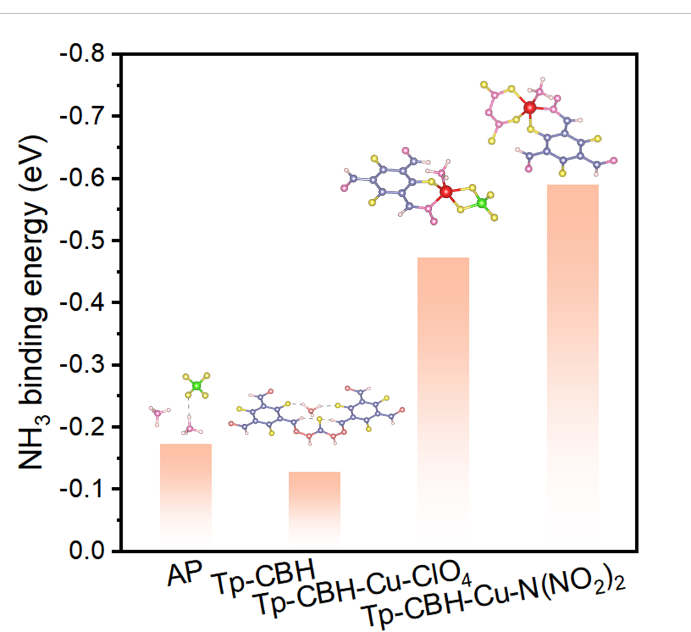


Figure S27. Comparison of NH_3_ bind energy of Tp-CBH, Tp-CBH-Cu-ClO_4_, and Tp-CBH-Cu-N(NO_2_)_2_

Table S3. EXAFS fitting parameters at the Cu K-edge for various samples

| Sample | Shell | CN^a^ | R(Å)^b^ | σ^2^(Å^2^)^c^ | ΔE_0_(eV)^d^ | K-range/Å^-1^ | R-range/Å | R factor |
| --- | --- | --- | --- | --- | --- | --- | --- | --- |
| Cu foil | Cu-Cu | 12.0* | 2.54±0.01 | 0.0091 | 4.2±0.6 | 3.0-13.0 | 1.5-3.0 | 0.0024 |
| CuO | Cu-O | 3.8±0.4 | 1.95±0.01 | 0.0036 | 0.1±1.3 | 2.0-13.2 | 1.0-3.3 | 0.0150 |
|  | Cu-Cu | 4.8±1.0 | 2.92±0.01 | 0.0074 | 4.0±1.9 |  |  |  |
|  | Cu-Cu | 5.1±1.2 | 3.12±0.01 |  |  |  |  |  |
| Cu_2_O | Cu-O | 2.2±0.1 | 1.87±0.01 | 0.0049 | 7.2±0.8 | 2.0-12.0 | 1.0-3.5 | 0.0198 |
|  | Cu-Cu | 13.8±1.5 | 3.03±0.01 | 0.0186 |  |  |  |  |
| CuPc | Cu-N | 3.9±0.2 | 1.94±0.01 | 0.0033 | 1.2±1.4 | 3.0-13.0 | 1.2-3.0 | 0.0162 |
|  | Cu-N-C | 6.7±0.8 | 2.95±0.01 | 0.0041 |  |  |  |  |
| **Tp-CBH-Cu-ClO_4_** | **Cu-O** | **3.9±1.0** | **1.97±0.02** | **0.0060** | **2.9±3.4** | **3.0-11.1** | **1.0-3.0** | **0.0247** |
|  | **Cu-N** | **2.1±0.3** | **2.09±0.02** |  |  |  |  |  |
|  | **Cu-N-C** | **3.2±0.9** | **3.03±0.03** | **0.0110** | **11.6±4.7** |  |  |  |
| **Tp-CBH-Cu-N(NO_2_)_2_** | **Cu-O** | **3.5±0.3** | **1.90±0.01** | **0.0034** | **-2.3±1.5** | **3.0-11.4** | **1.0-3.5** | **0.0197** |
|  | **Cu-N** | **2.1±0.4** | **1.99±0.00** |  |  |  |  |  |
|  | **Cu-N-C** | **2.5±0.8** | **2.85±0.03** | **0.0060** | **3.6±7.0** |  |  |  |

1. CN, coordination number; (b) R, distance between absorber and backscatter atoms; (c) σ^2^, Debye-Waller factor to account for both thermal and structural disorders; (d) ΔE(0), inner potential correction; R factor indicates the goodness of the fit. S_0_^2^ was fixed to 0.802, according to the experimental EXAFS fit of Cu foil by fixing CN as the known crystallographic value. A reasonable range of EXAFS fitting parameters: 0.700 < Ѕ_0_^2^< 1.000; CN > 0; σ^2^Å^2^＞0; |ΔE_0_|< 15 eV; R factor < 0.02.

Table S4. Comparison of the peak temperature and heat release during the decomposition stages of AP, Cu(ClO_4_)_2_, Tp-CBH-Cu-ClO_4_, and Tp-CBH-Cu-N(NO_2_)_2_

| Sample | Heating rate (^o^C/min) | Crystal phase transition  (^o^C) | Low-temperature decomposition (^o^C) | High-temperature decomposition ( ^o^C) | | Low-temperature exothermic release (J/g) | | High-temperature exothermic release (J/g) | |
| --- | --- | --- | --- | --- | --- | --- | --- | --- | --- |
| AP | 5 | 243.6 | 301.6 | 410.3 | 155 | | 483 | |  |
| Cu(ClO_4_)_2_ | 5 | 243.6 | 258.5 | 347.2 | 277 | | 719 | |  |
| Tp-CBH-Cu-ClO_4_ | 5 | 243.6 | - | 351.3 | - | | 1069 | |  |
| Tp-CBH-Cu-N(NO_2_)_2_ | 5 | 243.6 | - | 341.3 | - | | 1086 | |  |
| Tp-CBH-Cu-OAc | 5 | 243.6 | - | 365.2 | - | | 870 | |  |
| Tp-CBH-Cu-Cl | 5 | 243.6 | - | 364.5 | - | | 794 | |  |

Table S5. Peak temperatures and thermodynamic parameters of AP, Tp-CBH-Cu-ClO_4_, and Tp-CBH-Cu-N(NO_2_)_2_ at different heating rates

| Sample | β (℃/min) | | | | Kinetic parameters | | |
| --- | --- | --- | --- | --- | --- | --- | --- |
|  | 5 | 10 | 15 | 20 | logA_k_ | E_k_ (KJ/mol) | R_k_ |
| AP (HTD) | 410.3 | 419.0 | 425.0 | 431.3 | 17.31 | 255.8 | 0.9935 |
| Tp-CBH-Cu-ClO_4_ | 351.3 | 362.5 | 368.7 | 373.1 | 14.64 | 202.4 | 0.9997 |
| Tp-CBH-Cu-N(NO_2_)_2_ | 341.3 | 352.6 | 360.7 | 364.3 | 13.07 | 181.1 | 0.9981 |

Part 3. Supplementary References

1. Ravel, B.; Newville, M. ATHENA, ARTEMIS, HEPHAESTUS: data analysis for X-ray absorption spectroscopy using IFEFFIT. *J. Synchrotron Radiat.* **12**, 537-541 (2005).
2. Zabinsky, S. I.; Rehr, J. J.; Ankudinov, A.; Albers, R. C.; Eller, M. J. Multiple-Scattering Calculations of X-Ray-Absorption Spectra. *Phys. Rev. B* **52**, 2995-3009 (1995).
3. Kresse, G. From ultrasoft pseudopotentials to the projector augmented-wave method. *Phys. Rev. B* **59**, 1758-1775 (1999).
